# Humidity-Enhanced Direct Air Capture of Carbon Dioxide Using Amine-Grafted Covalent Organic Frameworks Under Ambient and Sub-ambient Temperatures

**DOI:** 10.1021/acs.chemmater.5c02891

**Published:** 2026-03-10

**Authors:** Arkaprabha Giri, Jiaqi Zhang, Xin Deng, Christopher W. Jones

**Affiliations:** School of Chemical & Biomolecular Engineering, 1372 Georgia Institute of Technology, Atlanta, Georgia 30332, United States

## Abstract

The significant rise in atmospheric CO_2_ and its impact on accelerating climate change have triggered intense efforts to develop porous sorbents for direct air capture (DAC), a route toward carbon-neutrality. Amine-functionalized covalent organic frameworks (COFs), an emerging class of crystalline porous materials, have recently shown promising potential for DAC at ambient, indoor temperatures (25 °C). However, most of Earth’s land area has annual mean temperatures below 25 °C, accompanied by nonzero and variable relative humidity (RH). The performance of amine-grafted COFs under cold, humid conditions remains largely unexplored, even though such climates represent the majority of potential DAC deployment sites. Herein, we report a systematic investigation of a tetrahydroquinoline-linked COF covalently functionalized with diverse amines, evaluating its CO_2_ adsorption behavior across a broad range of ambient to sub-ambient temperatures (25 °C to −20 °C) and relative humidities (0%–70%). A unique tris­(2-aminoethyl)­amine-functionalized COF (ImCOF-TAEA) achieved a pseudoequilibrium capacity of 0.46 ± 0.02 mmol g^–1^ under dry conditions, rising ∼137% to 1.09 ± 0.09 mmol g^–1^ under 70% RH using 400 ppm of CO_2_ at 25 °C. Upon cooling to 15 °C under 70% RH, the uptake further increased to 1.25 ± 0.02 mmol g^–1^, showing a 205% enhancement relative to dry conditions. *In situ* spectroscopic analysis supports the mechanism behind the unusually high enhancement in CO_2_ adsorption under humid conditions. ImCOF-TAEA also demonstrates excellent recyclability under ambient/sub-ambient conditions and has modest (45 °C-65 °C) requirements for regeneration.

## Graphical abstract

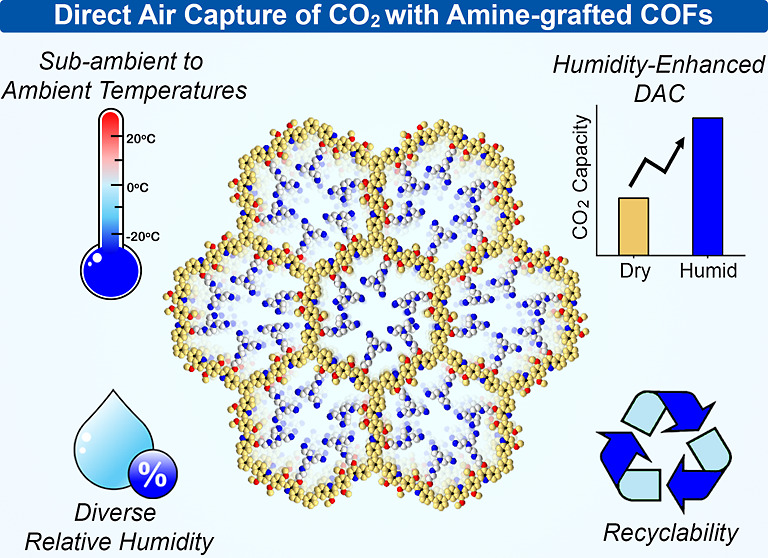

The anthropogenic CO_2_ emissions due to continued fossil fuel combustion have increased the atmospheric CO_2_ concentration from 280 ppm in the pre-industrial era to ∼430 ppm in 2025, according to the National Oceanic and Atmospheric Administration (NOAA).^1^ This sharp increase in CO_2_ level has driven a global temperature increase of ∼1.5 °C relative to the preindustrial era, resulting in pronounced change in weather patterns, such as frequent storms, extended heat waves, widespread wildfires, severe flooding, sea level rising, etc., which have severely impacted the ecosystems and biodiversity.^2–4^ While considerable efforts have been made to reduce dependence on fossil fuels by promoting the use of renewable energy sources, such strategies are only slowing the CO_2_ emission rate rather than eliminating excess CO_2_ in the atmosphere. Therefore, the deployment of negative emissions technologies (NETs) has become essential.^5,6^ In this context, direct air capture (DAC),^7–10^ a method that removes CO_2_ directly from ambient air, has recently gained substantial attention. Extensive research has been conducted across the world to design advanced sorbent materials that selectively capture CO_2_ from the atmosphere with a high uptake capacity, energy-efficient regeneration, and sufficient stability. When combined with geologic storage of the captured CO_2_, DAC can be a negative emission technology.

Among the various solid-state sorbents for DAC,^11^ amine-functionalized materials such as zeolites,^12–14^ silica,^15,16^ alumina,^17^ metal oxides,^18^ and metal–organic frameworks (MOFs),^19–21^ have shown promising performance under ambient temperature conditions, having high uptake capacity, good CO_2_ selectivity, and mild regeneration conditions. Recently, covalent organic frameworks (COFs),^22–32^ a class of crystalline porous organic polymers with large surface area, tractable pore sizes, high structural and functional tunability, and excellent physicochemical stability have emerged as attractive candidates for DAC. Yaghi and co-workers demonstrated several strategies for grafting polyamines onto COFs that showed high CO_2_ uptake capacity, low-temperature regeneration, and excellent cycling stability.^33–35^ However, most studies to date focus on evaluating the aforementioned materials at ambient and above-ambient temperatures (>20 °C).^17,36^ To the best of our knowledge, amine-functionalized COFs have not yet been evaluated under sub-ambient temperatures in both dry and humid conditions. This is crucial for DAC, as sorbents must operate in all seasons and all-weather conditions (Figure 1a).

Because over 70% of the Earth’s land surface has an annual average temperature below 25 °C, and relative humidity over land rarely falls below 35% (Figure 1a),^17,36–38^ the preponderance of studies at indoor ambient temperature and dry conditions do not provide sufficient insight into adsorbent performance. Specifically, the lack of data on DAC performance at sub-ambient conditions (<20 °C), particularly under humid conditions, poses a major barrier to advancing DAC technologies and scaling them for real-world applications. Importantly, sub-ambient temperature conditions offer distinct advantages for DAC. The absolute humidity is much lower at cold temperatures, which may limit competitive water sorption and reduce the energy consumption for desorbing water during regeneration cycles.^36^ Furthermore, cold temperatures promote physisorption with lower enthalpies of adsorption that can potentially enable smaller temperature swings for regeneration, reducing the overall energy demand of the DAC process.^36^ Therefore, investigating the performance of adsorbents at sub-ambient temperatures is not only critical for the widespread deployment of DAC plants but also essential for improving the energy efficiency and overall viability of DAC systems.

**Figure 1:**
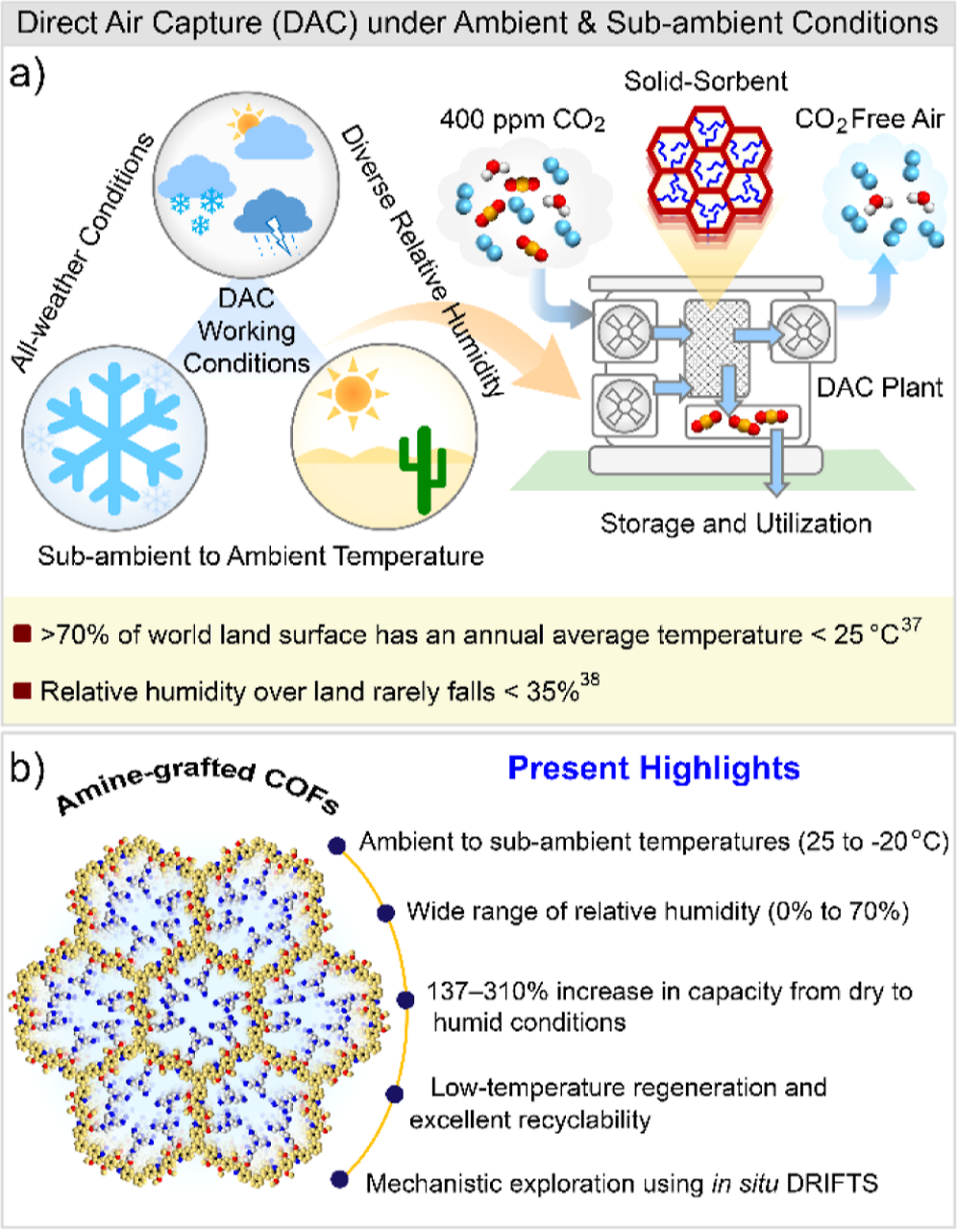
(a) Pictorial illustration of the various operating conditions for direct air capture (DAC), including all-weather and all-season environments, spanning temperatures from ambient to sub-ambient, and covering a broad range of relative humidities. (b) Present study highlighting amine functionalized COFs explored for DAC under diverse humidity and ambient and sub-ambient conditions.

Previous studies have shown that amine-functionalized sorbents, such as oxides, MOFs display contrasting CO_2_ capture behaviors from ambient to sub-ambient temperatures, driven by multiple enthalpic and entropic factors.^17,18,36^ These contrasting observations motivate us to systematically study amine-functionalized all-organic sorbents, like COFs, across sub-ambient to ambient temperatures under varying relative humidities. In this study, we post-synthetically functionalized a tetrahydroquinoline-linked COF (ImCOF-Cl) with a series of polyamines, such as tetraethylenepentamine (TEPA) and tris­(2-aminoethyl)­amine (TAEA) that have not been previously explored in COF systems. We systematically evaluated their performance for CO_2_ capture from simulated air (∼400 ppm of CO_2_/N_2_) under both ambient and sub-ambient temperatures (25 °C to −20 °C) and varying relative humidity levels (0% to 70% RH, Figure 1b). Among the materials developed, the TAEA-functionalized COF, i.e., ImCOF-TAEA, exhibited the most promising performance, achieving pseudoequilibrium CO_2_ uptake capacities (95% of inlet CO_2_ concentration, *C*_0_ = 400 ppm) exceeding 1 mmol g^–1^ between −5 and 25 °C under humid conditions, with the highest uptake 1.25 ± 0.2 mmol g^–1^ at 15 °C, 70% RH. Moreover, presaturating ImCOF-TAEA with water at 15 °C and 70% RH prior to CO_2_ adsorption yielded an uptake of 1.52 mmol g^–1^. Therefore, the presence of water significantly enhanced CO_2_ adsorption of the amine-functionalized ImCOFs (ImCOF-TAEA, ImCOF-TEPA), with uptake increased up to ∼310% under humid conditions compared to dry conditions. This humidity-assisted enhancement in CO_2_ capacity was further elucidated using *in situ* diffuse reflectance infrared Fourier transform spectroscopy (DRIFTS), providing insights into the underlying adsorption mechanism. Moreover, ImCOF-TAEA demonstrated efficient low-temperature regeneration (45–65 °C) and excellent cycling stability over multiple temperature swing adsorption–desorption cycles under ambient (25 °C) and sub-ambient conditions (15 °C). These preliminary findings highlight the potential of amine-grafted COFs, as effective and energy-efficient adsorbents for direct air capture applications under cold humid environmental conditions.

## Results and Discussion

### Design and Synthesis

The amine-grafted COFs were synthesized by the post-synthetic modification of an imine-linked COF with 2-chloroethoxy tetrahydroquinoline linkages followed by installation of different amines (Figure 2a and Section S1). The basic skeleton of the COF (ImCOF) was synthesized by the aqueous acetic acid catalyzed Schiff base condensation between a tridentate amine, 1,3,5-tris­(4-aminophenyl)­benzene (TAPB) and bidentate aldehyde, 2,5-dimethoxyterephthalaldehyde (DMTP).^39^ Next, the imine linkages of ImCOF were converted to 2-chloroethoxy tetrahydroquinoline linkages through a FeCl_3_ catalyzed aza Diels–Alder cycloaddition reaction with 2-chloroethyl vinyl ether to obtain the tetrahydroquinone (THQ)-linked COF, ImCOF-Cl.^33^ Finally, the grafting of different amines, such as tris­(2-aminoethyl)­amine (TAEA), and tetraethylenepentamine (TEPA) was performed by the nucleophilic substitution reaction of Cl in chloroethoxy groups of ImCOF-Cl followed by washing with NaOH (0.5 M in methanol), ammonium hydroxide solution, and different organic solvents to obtain ImCOF-amines, such as ImCOF-TAEA, ImCOF-TEPA (Figure 2a).

**Figure 2:**
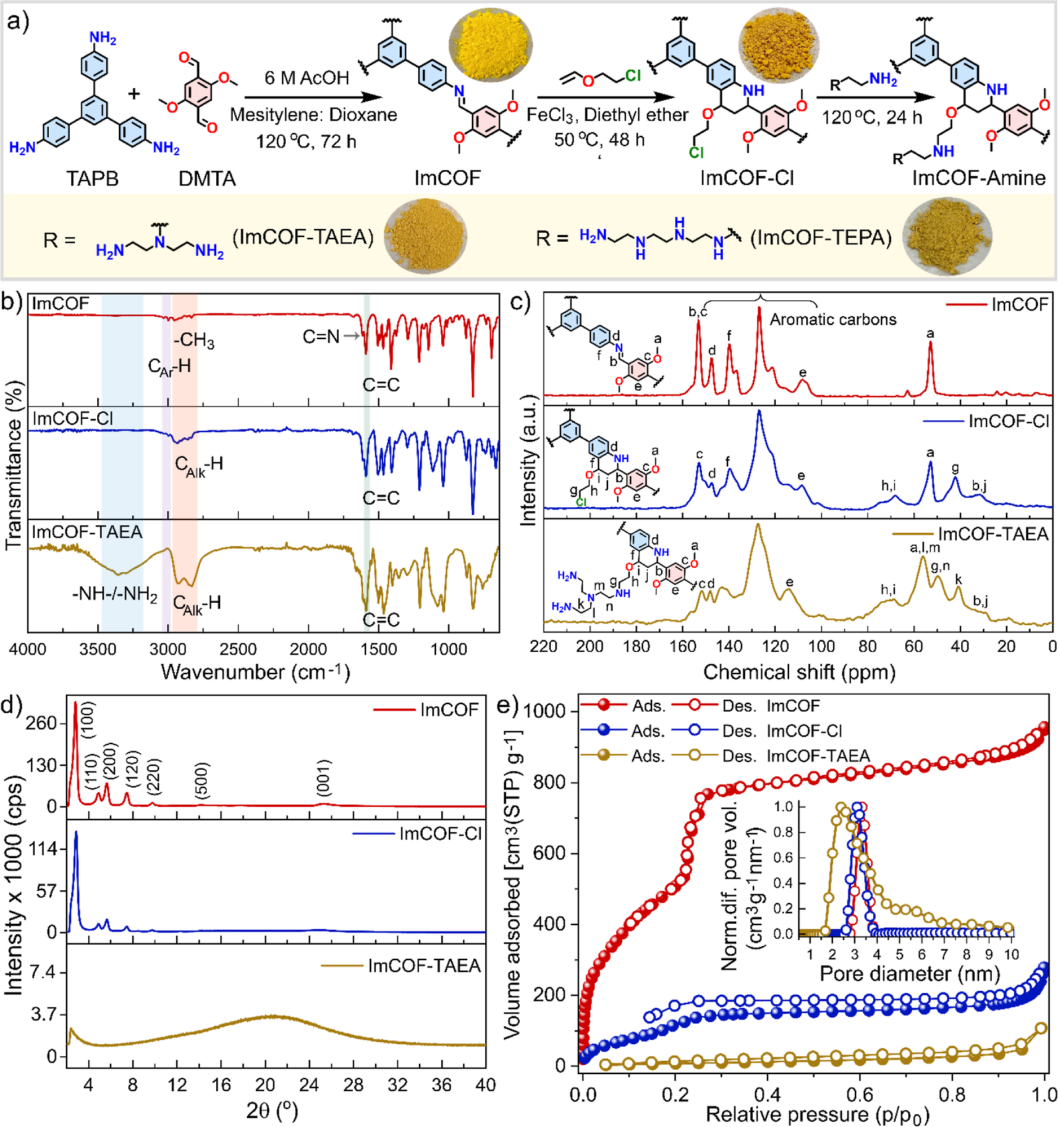
(a) Schematic representation of the stepwise synthesis of amine-functionalized covalent organic frameworks (COFs). Digital images of ImCOF, ImCOF-Cl, ImCOF-TAEA, and ImCOF-TEPA. (b) Fourier transform infrared (FTIR) spectra of ImCOF, ImCOF-Cl, and ImCOF-TAEA. (c) Stacked solid state ^13^C CP/MAS NMR spectra of ImCOF, ImCOF-Cl, and ImCOF-TAEA. (d) Powder X-ray diffraction (XRD) patterns of ImCOF, ImCOF-Cl, and ImCOF-TAEA. (e) Nitrogen sorption isotherms of ImCOF, ImCOF-Cl, and ImCOF-TAEA at 77K (inset: QSDFT pore size distribution of the respective materials).

To understand the stepwise conversion process, we conducted various spectroscopic and textural characterizations of the materials after each step (Figures 2b–d and S1–S11). Upon crystallization, the Fourier transform infrared (FTIR) spectrum of ImCOF was first compared to the spectra of the starting monomers, TAPB and DMTA (Figures 2b and S1). The vibrational absorbance bands at 1668 cm^–1^ for the aldehyde C=O stretch observed in DMTA and the absorbance bands at around 3434, 3354, and 3209 cm^–1^ for the amine N–H stretches in TAPB were largely attenuated in the spectrum of ImCOF. A new peak at 1615 cm^–1^ arises, suggesting the successful formation of the imine linkage.^39^ In the following step, i.e., aza-Diels–Alder reaction to obtain ImCOF-Cl, the absorbance at 1615 cm^–1^ for the imine C=N stretching in ImCOF was shifted to 1610 cm^–1^. The emergence of broad bands at 2937 to 2835 cm^–1^ in ImCOF-Cl indicated the introduction of aliphatic C–H bonds as part of the as-synthesized tetrahydroquinoline (THQ) linkage and chloroethoxy side chain. In the last step, the higher intensity of the absorbance bands at 2930 and 2830 cm^–1^ indicated the introduction of aliphatic amines in ImCOF-amines, e.g., ImCOF-TAEA and ImCOF-TEPA, through nucleophilic substitution, which was also confirmed by the broad absorbance band at 3350 cm^–1^ (N–H stretch, Figures 2b and S1). Thermogravimetric analysis (TGA) suggested ImCOF is thermally stable up to 380 °C under air (and 400 °C under N_2_, Figure S2), whereas, amine-grafted COFs are stable up to 200 °C under air and N_2_ (Figure S2). The lower onset temperature reflects the expected thermal decomposition of the grafted amine functionalities, while the COF skeleton retains its high inherent stability, as observed from differential TGA (Figure S2c).

To further confirm the two-step modification process, we carried out the solid-state ^13^C cross-polarization magic angle spinning (CP/MAS) NMR analysis (Figures 2c and S3). The peak at 153 ppm of ImCOF is attributed to both the imine carbon and the aromatic carbon attached to the methoxy group. This peak intensity in ImCOF-Cl is greatly diminished, whereas several new peaks in the range of 20–80 ppm appear, due to the aliphatic carbon associated with the THQ linkage and the chloroethoxy side chain. Moreover, ImCOF and ImCOF-Cl demonstrate distinctive chemical compositions as shown by solid-state ^1^H–^13^C heteronuclear correlation (HETCOR) NMR spectra (Figure S4). In the aromatic region, the signal correlating ^13^C at 155 ppm in ImCOF indicates the imine C–H correlation, which disappears in the case of ImCOF-Cl due to the formation of the THQ moiety.^33,39^ Another signal at 140 ppm disappears after the transformation to ImCOF-Cl, which is assigned to carbon “f” losing hydrogen through the THQ formation (Figures 2c and S4). Multiple correlations of C–H from the aromatic skeleton are shown in the 120–126 ppm region. In the aliphatic region, the methoxy groups are present in both cases, with a slight shift from around 53 to 55 ppm. The signals at around 74, 69, 44, 38 ppm are assigned to the 2-chloroethyl vinyl ether, with a weak signal at ∼30 ppm indicating the methylene moiety (assigned as “j”) in the THQ (Figures 2c and S4). After amine functionalization (ImCOF-TAEA, ImCOF-TEPA), several new peaks emerge in solid-state ^13^C (CP/MAS) NMR in the range of 20–80 ppm, showcasing the successful integration of polyamines in the framework (Figures 2c and S3).

The close agreement between the theoretical and the experimental CHN values in elemental analysis of ImCOF indicates the high purity of the material (Table S1). The deviation in experimental Cl content (expt: 8.4 wt %, theor: 11.7 wt %) in ImCOF-Cl suggests the partial retention of imine linkages is favorable for preserving crystallinity and porosity after post-modification. The absence of Cl in ImCOF-TAEA indicates complete amine functionalization of ImCOF-Cl. It is noteworthy that the degree of amine functionalization, estimated from the N content, varies slightly across the different ImCOF-amines: ImCOF-TAEA (12.9 ± 0.3 wt %): ∼78%, ImCOF-TEPA (13.4 ± 0.3 wt %): ∼71%. The lower degree of amine installation in the latter case is attributed to some amines cross-linking with adjacent Cl atoms in ImCOF-Cl. Field emission scanning electron microscopy (FE-SEM) images of the ImCOF and ImCOF-Cl suggest a homogeneous distribution of aggregated particles (Figure S7a,b). The FESEM images revealed that the morphology of amine functionalized COFs, e.g., ImCOF-TAEA and ImCOF-TEPA, remain almost unaltered during the post-synthetic modifications (Figure S7c,d). Elemental mapping of ImCOF-TAEA and ImCOF-TEPA further confirms almost all the –Cl groups were replaced by amines (Figure S8).

The powder X-ray diffraction (PXRD) pattern of ImCOF showed prominent peaks at 2.8°, 4.9°, 5.6°, 7.5°, 9.8°, 14.1°, and 25.3°, corresponding to the (100), (110), (200), (210), (220), (500), and (001) Miller planes, respectively, indicating an eclipsed (AA) stacking mode, consistent with literature (Figures 2d and S5a,b).^39^ After post-synthetic modifications of ImCOF through the aza-Diels–Alder reaction, the peak position did not change. Pawley refinement of the experimental PXRD pattern led to the following unit cell parameters: *a* = 37.36 Å, *b* = 36.99 Å, *c* = 4.96 Å; α = β = 90°, γ = 120°, with *R*_p_: 3.0% and *R*_wp_: 4.0%, Figures 2d and S5c,d. However, the nonplanarity of the tetrahydroquinoline linkages and the possible presence of stereoisomers contributed to the reduced crystallinity observed in ImCOF-Cl compared to ImCOF.^33^ Furthermore, chemical functionalization with different amines led to a loss of crystallinity in the resulting COFs (ImCOF-TAEA, ImCOF-TEPA) due to the random orientations of flexible polyamines within the pores, a trend consistently observed in amine-functionalized COFs (Figures 2d and S6).^33–35^

The N_2_ sorption isotherm measurements on ImCOF at 77 K revealed a specific Brunauer–Emmett–Teller (BET) surface area of 2033 ± 22 m^2^ g^–1^, with a pore size of 3.3 nm [according to quenched solid density functional theory (QSDFT) model] and pore volume of 1.35 ± 0.02 cm^3^ g^–1^ (at *P*/*P*_0_ = 0.95), which is in good agreement with the modeled pore size of the AA stacking mode (Figures 2d,e and S9–S11, Table S2).^39–43^ The specific BET surface area of ImCOF-Cl was found to be 376 ± 54 m^2^ g^–1^ with slight reduction in the pore size (3.1 nm). The reduced BET surface area is attributed to the increased framework mass and decreased pore volume. After amine installation, the surface areas further decreased, with values of 52 ± 10, and 37 m^2^ g^–1^ for ImCOF-TAEA, and ImCOF-TEPA, respectively, due to amine incorporation (Figures 2e and Table S2). The Barrett–Joyner–Halenda (BJH) and QSDFT models suggested the pore size remained concentrated in the narrow mesoporous region (2.4–3.5 nm), indicating the preservation of the 1D pore channels of COF after amine functionalization (Figures 2e and S10a). Further, CO_2_ sorption analysis at 273 K was employed to probe the ultramicropore domain of the amine-functionalized COFs, revealing a dominant pore at ∼0.35 nm [nonlocal density functional theory (NLDFT) model], which can be attributed to the interlayer spacing of 2D COF layers (Figure S10b).^39^

### CO_2_ and Water Adsorption Isotherms

The CO_2_ adsorption capacities of amine-grafted COFs were initially evaluated through thermogravimetric analysis (TGA) by exposing amine functionalized COF samples under 400 ppm of CO_2_/N_2_ for 12 h at 30 °C dry conditions (Figures 3a and S12). TGA data indicated 0.44 ± 0.06 and 0.27 ± 0.06 mmol g^–1^ uptake capacities for ImCOF-TAEA and ImCOF-TEPA, respectively. ImCOF-TEPA requires approximately 30 min to reach 50% of its total uptake and 163 min to reach 80% capacity. In contrast, ImCOF-TAEA exhibits faster adsorption performance, achieving 50% and 80% of its total CO_2_ capacity within 20 and 129 min, respectively (Figure S12b). We also conducted TGA under 10% CO_2_ (comparable to flue gas conditions) at 25 °C under dry conditions, where ImCOF-TAEA and ImCOF-TEPA showed considerably higher uptakes of 1.35 ± 0.15 and 1.32 ± 0.08 mmol g^–1^, respectively (Figure S12). Although both the COFs have similar N-content (ImCOF-TAEA: 9.21 ± 0.21 mmol g^–1^; ImCOF-TEPA: 9.6 ± 0.2 mmol g^–1^), the difference in DAC capacity may stem from the distinct amine compositions present in the COFs. Previous studies have shown that primary amines exhibit a higher CO_2_ affinity than secondary amines at comparable amine loadings, while tertiary amines are essentially inactive under dry DAC conditions.^44,45^ TAEA provides two primary and one secondary amines per molecule, whereas TEPA offers only one primary and four secondary amines (Figure S13). Elemental analysis further suggests a greater degree of cross-linking in ImCOF-TEPA, reducing its primary/secondary amine content and likely contributed to its lower DAC performance compared to ImCOF-TAEA. In contrast, at higher CO_2_ concentrations (10% CO_2_/N_2_), where both primary and secondary amines contribute comparably to CO_2_ binding, the two materials showed considerably higher, nearly similar uptakes.

**Figure 3:**
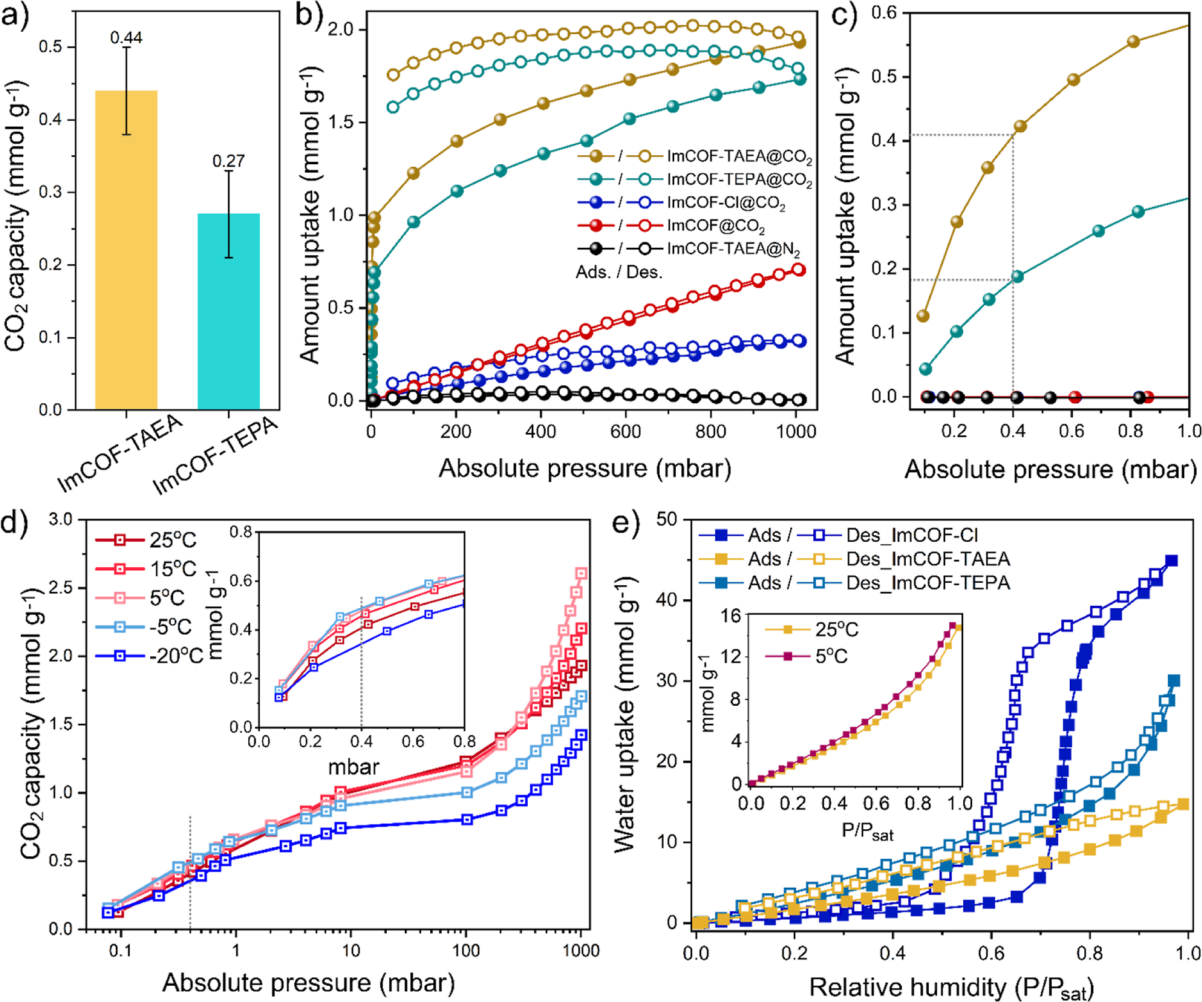
(a) CO_2_ uptake capacities obtained from thermogravimetric analyses (TGA) of ImCOF-TAEA and ImCOF-TEPA under the exposure of 400 ppm of CO_2_/N_2_ for 12h at 30 °C. (b) Single-component CO_2_ and N_2_ sorption isotherms of ImCOF, ImCOF-Cl, ImCOF-TAEA, and ImCOF-TEPA at 25 °C. (c) Zoomed-in view of the isotherms highlighting the uptake at the ambient CO_2_ pressure (0.4 mbar). (d) Single-component CO_2_ sorption isotherms of ImCOF-TAEA at different temperature (−20 to 25 °C), (e) water vapor adsorption isotherms of ImCOF-Cl, ImCOF-TAEA and ImCOF-TEPA, inset: water vapor adsorption isotherms of ImCOF-TAEA at 25 and 5 °C.

The CO_2_ adsorption isotherms of ImCOF-TAEA and ImCOF-TEPA at 25 °C showed a sharp rise at CO_2_ pressures below 1.0 mbar, followed by a gradual increase at higher pressures (Figure 3b,c). Hysteresis in the adsorption–desorption isotherms was indicative of strong chemical interactions between amine-functionalized COFs and CO_2_.^35^ Based on the single-component adsorption data, the CO_2_ capacity of ImCOF-TAEA was found to be 0.41 mmol g^–1^ at 0.4 mbar (400 ppm, concentration close to the CO_2_ pressure at atmospheric conditions) at 25 °C. The negligible N_2_ uptake of ImCOF-TAEA suggests its high CO_2_ selectivity over N_2_ at 25 °C (isotherm with black filled and hollow circles, Figure 3a).^34^ The equilibrium uptake capacity further increased from 0.41 to 0.49 mmol g^–1^ upon decreasing the temperature from 25 °C to −5 °C, respectively (Figure 3d). However, further decreasing the temperature from −5 °C to −20 °C, lowered the uptake capacity to 0.35 mmol g^–1^. The decline in CO_2_ uptake capacity at lower temperatures can be attributed to the reduced thermal energy at these conditions, which restricts amine chain mobility and limits their ability to efficiently interact with CO_2_ molecules.^46–48^

Humidity has a strong influence on the DAC performance.^18,19,49^ While high water uptake by the adsorbents can increase the energy consumption for desorbing water during regeneration, an optimal level of water adsorption is often essential to facilitate efficient CO_2_ capture. To estimate the water uptake capacity and also probe the pore environment (hydrophobic or hydrophilic) of ImCOF-Cl before and after amine functionalization, we performed water vapor adsorption isotherm analysis (Figures 3e and S14).^50^ ImCOF-Cl exhibited a Type V isotherm with increased water uptake at higher RH (*P*/*P*_0_ > 0.7), suggesting weak water–adsorbent interactions at lower RH due to its hydrophobic pore environment followed by capillary condensation at higher relative pressures. Meanwhile, amine-grafted ImCOFs, such as ImCOF-TAEA and ImCOF-TEPA, showed characteristic of Type III isotherms, where adsorption on the surface is initially weak, becoming more favorable as coverage increases. The water uptake capacities of ImCOF-TAEA at 25 °C, were found to be 2.6, 4.7, and 7.3 mmol g^–1^ at 30%, 50%, and 70% RH, respectively (Figure 3e). At lower temperatures, such as 5 °C, the water uptake of ImCOF-TAEA was slightly increased to 2.9, 5.3, and 8.4 mmol g^–1^ at 30%, 50%, and 70% RH, respectively. The isosteric heat of water vapor adsorption was estimated to be 46–50 kJ mol^–1^ (Figure S15). ImCOF-TEPA also showed low water uptake of 3.8, 7.0, and 11.2 mmol g^–1^ at 30%, 50%, and 70% RH (25 °C), respectively (Figure 3e). The water uptake capacities of ImCOF-amines are comparable or relatively lower than many amine functionalized benchmark chemisorbents for DAC, including TEPA-impregnated γ-Al_2_O_3_ (12 mmol g^–1^ at 70% RH),^48^ TEPA-impregnated Mg_2_(dobpdc) (14–16 mmol g^–1^),^51^ TEPA-impregnated MIL-101­(Cr) (24 mmol g^–1^ at 70% RH),^48^ PEI-impregnated Mg_0.55_Al–CO_3_ layered double hydroxides (LDHs), Mg_0.55_Al–O mixed metal oxides (MMOs) (18–20 mmol g^–1^ at 70% RH),^18^ and COF-999 (5 mmol g^–1^ at 50% RH),^34^ paving the way for low-energy input in the regeneration step upon cycling.

### CO_2_ Breakthrough Experiments

To further check the CO_2_ capture performance for practical DAC applications and to understand the effect of temperature and humidity on CO_2_ adsorption performance, we have carried out dynamic breakthrough experiments under different temperatures ranging from 25 °C to −20 °C with varying relative humidities from 0% to 70% (Figure 4 and Section S4.4). A breakthrough experiment was first conducted with an empty bed to determine the mean residence time of the gas stream through the fixed bed (gray curve designated as background, Figure 4a). Pseudoequilibrium capacities were determined from the breakthrough curves in Figure 4a at the time points when 95% of *C*_0_ was reached, with the results for both dry and humid conditions at 25 °C also presented in Figure 4b. With gradually increasing relative humidity from 0% to 70% at 25 °C, the CO_2_ breakthrough time increases. The pseudoequilibrium capacity of ImCOF-TAEA was found to be 0.46 ± 0.02, 0.53 ± 0.07, 0.76 ± 0.03, 1.09 ± 0.06 mmol g^–1^ for 0%, 30%, 50% and 70% RH, respectively (Figure 4b). For ImCOF-TEPA, the capacity increased from 0.21 mmol g^–1^ to 0.86 mmol g^–1^ by switching the RH from 0% to 70% at 25 °C (Figure S16). Switching from 0% to 70% RH at 25 °C led to a remarkable increase in CO_2_ uptake, ∼137% for ImCOF-TAEA and ∼310% for ImCOF-TEPA, which, to the best of our knowledge, represents one of the highest reported enhancements among the chemically grafted amine sorbents with increasing humidity (Table S4).^35^

**Figure 4:**
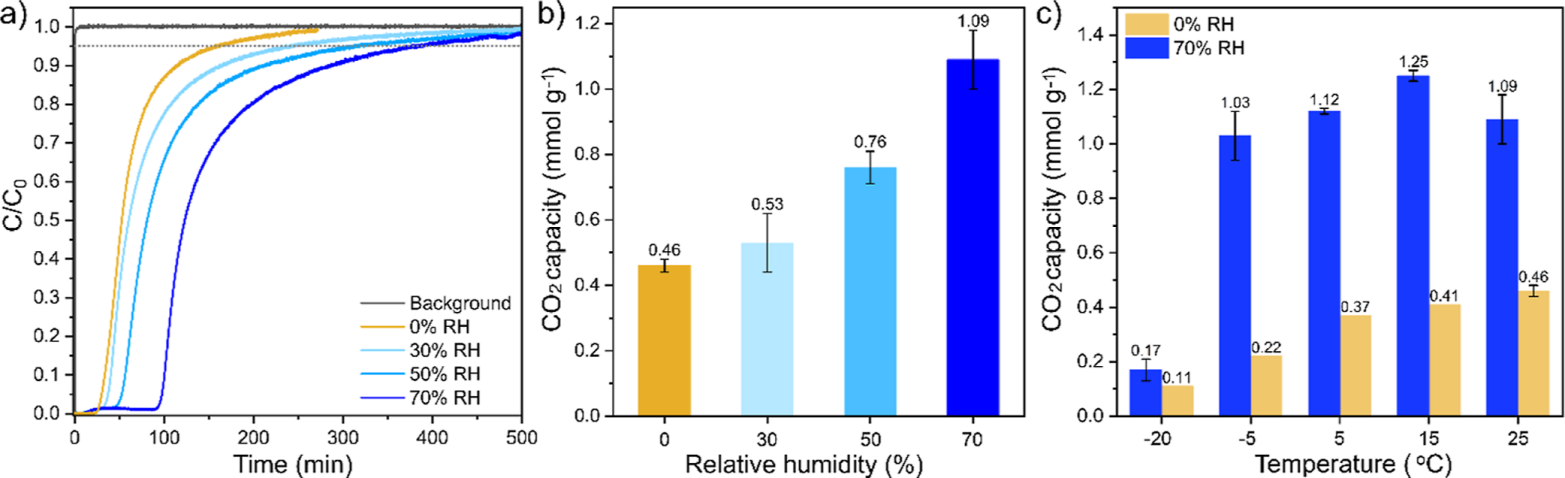
(a) CO_2_ dynamic breakthrough curves and (b) estimated CO_2_ uptake (pseudoequilibrium capacity, *C*/*C*_0_ = 0.95) under 400 ppm of CO_2_/N_2_ with 0%, 30%, 50%, and 70% relative humidity (RH) at 25 °C for ImCOF-TAEA. (c) Pseudoequilibrium CO_2_ uptake capacity of ImCOF-TAEA at diverse ambient and sub-ambient temperatures under dry (0% RH) and humid (70% RH) conditions.

### Temperature Strongly Influences CO_2_ Sorption Behavior

Lower temperatures thermodynamically favor both physisorption and the exothermic chemisorption of CO_2_ by amines. However, chemisorption becomes kinetically and configurationally constrained at low temperatures due to reduced amine mobility required for effective CO_2_ binding. The resulting trade-off among these factors ultimately governs the CO_2_ uptake capacity of amine-based sorbents across different temperatures. Lowering the temperature from 25 to 15 °C, we found the pseudoequilibrium capacity of ImCOF-TAEA under 70% RH, increased from 1.09 ± 0.09 to 1.25 ± 0.02 mmol g^–1^ (Figures 4c and S17), reflecting favorable thermodynamic contributions. Further lowering the temperature from 15 to 5 °C and −5 °C under 70% RH, the CO_2_ capacity remained similar, i.e., 1.12 ± 0.01, and 1.03 ± 0.09 mmol g^–1^, respectively, indicating the onset of kinetic limitations. However, under dry conditions, the CO_2_ capacity gradually decreased from 0.46 to 0.22 mmol g^–1^ upon lowering the temperature from 25 °C to −5 °C (Figures 4c and S17a). At −20 °C, we observed that the CO_2_ uptake was significantly reduced under both dry and 70% RH conditions. The reduced CO_2_ uptake capacity of ImCOF-TAEA at lower temperatures can be attributed to the kinetically limited sorption due to decreased amine mobility and low absolute humidity (∼870 ppm). In contrast, when ImCOF-TAEA was presaturated with water prior to CO_2_ adsorption (at 70% RH), the pseudoequilibrium capacity increased to 0.45 and 1.52 mmol g^–1^ at −20 and 15 °C, respectively, compared to the coadsorption method (Figure S18). This indicates that prehumidification retains amine mobility even at subzero temperature, leading to higher uptake.

The CO_2_ desorption performance of a sorbent directly affects both the operational cost and the long-term stability of the material, as high-temperature desorption can induce thermal and oxidative degradation.^52,53^ To evaluate this, temperature-programmed desorption (TPD) experiments were performed using ImCOF-TAEA (Figure S19). After CO_2_ adsorption under different conditions, the temperature was ramped to 80 °C at 0.5 °C min^–1^. Following adsorption under dry and humid conditions (30–70% RH) at 25 °C, ImCOF-TAEA desorbed >90% of CO_2_ within the 45–65 °C temperature range. Under cold and humid conditions (−5 to 15 °C), the majority of CO_2_ and H_2_O was released around 45–55 °C and 15–20 °C, respectively (Figures S20 and S21). Thus, the comparatively low-temperature regeneration conditions highlight the potential of ImCOF-TAEA to reduce the energy requirements and overall cost of the DAC process.

### Mechanistic Investigation of CO_2_ Adsorption under Dry and Humid Conditions

The nature of the chemisorbed species formed under dry and humid conditions is strongly influenced by multiple factors, including the pore structure of the amine-functionalized host materials (surface area, pore size, pore volume, and chemical functionalities on the pore surface), the type of amines (primary, secondary, or tertiary), and also the CO_2_ concentration.^18,48,54–56^ In both ImCOF-TAEA and ImCOF-TEPA, we observed a record high increase in CO_2_ adsorption (400 ppm of CO_2_/N_2_) capacity, i.e., 2.4-fold and 4.1-fold respectively, when switching from dry to humid (70% RH) conditions at 25 °C. There are several plausible reasons for this humidity-induced enhancement in CO_2_ uptake capacity, such as, an increase in the formation of alkylammonium carbamate ion pair (R-NH_3_^+^···^–^OOC-NH-R), or the formation of species which involve 1:1 reaction stoichiometry between the amine and CO_2_, like carbamic acids (R-HN-COOH) and bicarbonates (R-NH_3_^+^···HCO_3_^–^).^56^

To elucidate the reason for the increased CO_2_ uptake under humid conditions compared to dry conditions, we conducted a systematic mechanistic study using *in situ* DRIFTS under controlled dry and humid environments. Prior to CO_2_ exposure, the samples (i.e., ImCOF-TAEA, ImCOF-TEPA) were activated inside the DRIFTS cell at 120 °C for 2 h under a 60 sccm N_2_ flow, cooled to 25 °C, and then exposed to dry or humid (75% RH) 400 ppm of CO_2_/N_2_ at a flow rate of 40 sccm. To clearly visualize the structural changes associated with CO_2_ adsorption, the spectrum of the activated sample was subtracted from the spectra collected after CO_2_ exposure for a certain period of time (Figures 5a,b and S22). In the case of ImCOF-TAEA, under dry conditions, we observed the emergence of a new peak at around 1700 cm^–1^, corresponding to the C=O stretching of carbamic acid (Figure 5a and Table S3).^56–58^ Peaks around 1556 (ν_as_), 1425 (ν_s_), and 1380 (ν_s_) cm^–1^ were attributed to the asymmetric and symmetric stretching vibrations of carbamate (COO^–^), while the band at 1311 cm^–1^ corresponded to NCOO^–^ skeletal vibrations.^56^ Additional peaks at 1650 (δ_as_), 1537 (δ_as_), and 1473 (δ_s_) cm^–1^ indicated the formation of ammonium (NH_3_^+^) ions of the zwitterionic alkyl ammonium carbamate pairs.^56^ These results confirm the coexistence of carbamic acid and alkylammonium carbamate ion pairs under dry conditions, also in good agreement with earlier observations.^18,33,35^ In our previous work, we demonstrated that the extent of pore filling (accounts for the degree of amine clustering) and the BET surface area-to-pore volume (SA/PV) ratio of the support (accounts for the probability of interactions between amine and support) could correlate with whether carbamic acid, ammonium carbamate, or both species form during CO_2_ capture by amine-functionalized sorbents (Figure S23).^18^ In the present case, ImCOF-Cl (support) exhibits an SA/PV of 1.34 ± 0.21 nm^–1^ with pore fillings of 70–75% (after amine grafting, i.e., ImCOF-TAEA and ImCOF-TEPA), placing them in the regime where formation of both carbamic acid and carbamate under dry conditions is expected, as indicated by the previously reported extent of pore filling (%) vs SA/PV (nm^–1^) plot (Figure S23).

**Figure 5:**
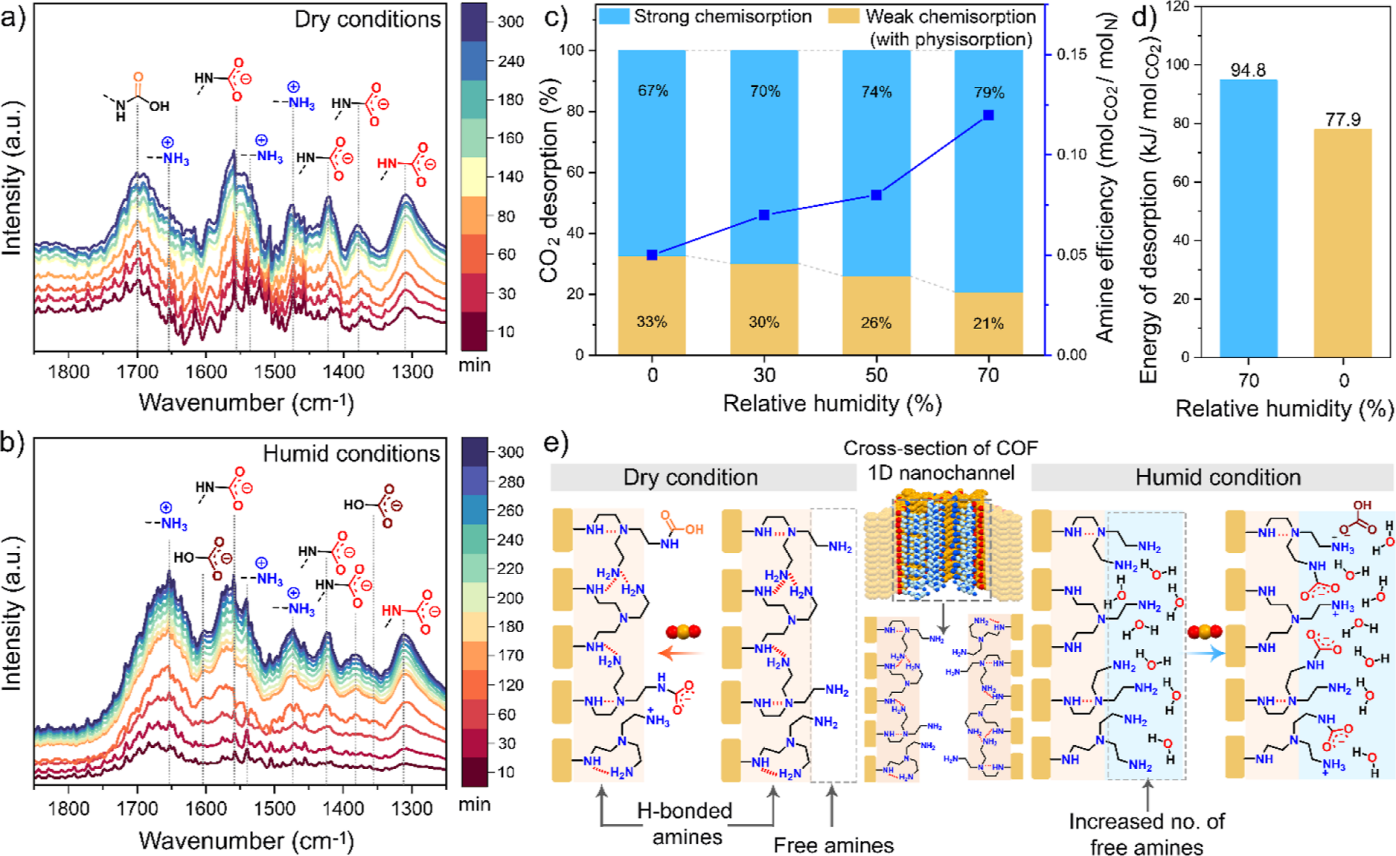
*In situ* DRIFT spectra of ImCOF-TAEA as a function of adsorption time at 25 °C under (a) dry and (b) humid conditions (75% RH). Yellow: carbamic acid, red: carbamate ion, blue: ammonium ion, brown: bicarbonate. Adsorption conditions: 400 ppm of CO_2_/N_2_; flow rate: 40 sccm; activation, 120 °C under 60 sccm N_2_ for 2 h. (c) Relative contribution (%) of weak chemisorption (including physisorption) and strong chemisorption in the total amount of desorbed dry/humid CO_2_ from ImCOF-TAEA during the temperature program desorption (TPD). Strong chemisorption (blue): CO_2_ desorption above 25 °C; weak chemisorption (yellow): CO_2_ desorption under 25 °C. (d) Energy of CO_2_ desorption under dry and humid (70% RH) conditions from ImCOF-TAEA. (e) Schematic illustration of CO_2_ adsorption mechanism by ImCOF-TAEA under dry and humid conditions.

In contrast, under humid conditions, no distinct peaks associated with carbamic acid were observed (Figure 5b). Instead, the dominant features were the peaks for R-NH_3_^+^ (1653, 1539, 1473 cm^–1^) and R-COO^–^ (1557, 1425, 1380, 1313 cm^–1^), confirming the predominant formation of alkylammonium carbamate ion pairs (Table S3). Notably, non-negligible peaks were also noticed in the longer time domain at around 1605 and 1355 cm^–1^, characteristic of bicarbonate species (HCO_3_^–^).^49,54,56^ However, the detection of HCO_3_^–^ on solid-supported amine sorbents under humid CO_2_ capture conditions is often challenging using FT-IR spectroscopy because of the low concentrations of bicarbonate formed and/or their weak IR absorption.^48,49^ Therefore, the presence of bicarbonates should be further verified through complementary techniques such as solid-state ^13^C NMR spectroscopy in future studies.^49,59–61^ The DRIFTS analysis for 400 ppm of CO_2_ capture using ImCOF-TEPA under the same dry and humid conditions also showed a similar trend as that of ImCOF-TAEA (Figure S22).

Alkyl ammonium carbamate ion pairs are associated with strong chemisorption and exhibit higher binding energies compared to carbamic acid and bicarbonate species, which are typically weak chemisorption products.^48,62,63^ To get an qualitative estimate for the contributions of weak chemisorption (including physisorption) and strong chemisorption to the overall CO_2_ uptake by ImCOF-TAEA under dry and humid conditions at 25 °C, we performed TPD experiments (Figures 5c and S19). The TPD experiments were conducted after exposing ImCOF-TAEA with 400 ppm of CO_2_/N_2_ at 25 °C under varying RH levels, using a temperature ramping rate of 0.5 °C min^–1^. Herein, CO_2_ desorption ≤25 °C is classified as weak chemisorption (including physisorption), while desorption above 25 °C is attributed to strong chemisorption. We observed that increasing the RH from 0% to 70% led to a rise in the contribution of strong chemisorption from 67% to 79% (Figures 5c and S19c). Simultaneously, the amine efficiency significantly improved from 0.05 to 0.12 mol CO_2_/mol N. These findings suggest that higher humidity levels promote higher extent of alkylammonium carbamate ion pairs formation. We have also estimated the energy of desorption (*E*_d_) of ImCOF-TAEA for the CO_2_ under dry and humid conditions based on a TPD method reported by Cvetanovic and Amenomiya (Figures 5d and S24).^48,64^ The calculated energy of desorption suggested that the binding energy of CO_2_ increases from 77.9 kJ mol^–1^ to 94.8 kJ mol^–1^ under dry and humid conditions, respectively. These results also support the stronger chemisorption under humid conditions due to preferential formation of alkylammonium carbamate ion pairs over carbamic acid (associated with weak chemisorption).

From the above experimental observations, we hypothesized that the substantial increase in CO_2_ adsorption capacity under humid conditions can be due to the preferential formation of alkylammonium carbamate ion pairs over carbamic acid, facilitated by humidity-enhanced amine accessibility (Figure 5e). Water frees the amines from strong amine–amine and amine-support interactions and thereby increases their accessibility toward CO_2_.^48,65,66^ As a result, a substantially larger fraction of amine sites participates in CO_2_ capture under humid conditions than under dry conditions. For example, the accessible amine content in ImCOF-TAEA increases by ∼2.5-fold at 25 °C under 70% RH compared to dry conditions (Section S4.7). Additionally, water promotes the formation and stabilization of the zwitterionic alkylammonium carbamate species by enhancing molecular mobility, reducing diffusion limitations, and enabling more efficient protonation of adjacent amine groups.^48,51,67,68^ Bicarbonate formation under humid conditions may further increase uptake due to theoretical 1:1 CO_2_-to-amine stoichiometry.^35^

### Recyclability

To evaluate the cycling stability of ImCOF-TAEA, we conducted both TGA and fixed-bed breakthrough experiments by applying consecutive adsorption–desorption temperature-swing cycles under simulated air (400 ppm of CO_2_/N_2_; Figure 6). In the TGA, using 400 ppm of CO_2_/N_2_ (flow rate: 90 sccm) and 0% RH, each cycle consisted of a 3 h adsorption at 25 °C followed by a 15 min regeneration step at 80 °C under N_2_ flow (flow rate: 90 sccm). Over 5 consecutive adsorption–desorption cycles, ImCOF-TAEA exhibited a stable CO_2_ uptake capacity of ∼0.5 mmol g^–1^ cycle^–1^ without any noticeable loss in the performance (Figure 6a). In fixed bed breakthrough studies, we have evaluated the recyclability of ImCOF-TAEA under ambient and sub-ambient temperatures (25 and 15 °C) with varying relative humidities (0% and 70%; Figures 6b and S25). Adsorption was terminated when the outlet CO_2_ concentration approached 95% of the equilibrium value (*C*_0_). After each adsorption cycle, regeneration of ImCOF-TAEA was carried out at 80 °C, under N_2_ flow (flow rate: 60 sccm) for 2h. Fixed-bed breakthrough studies on ImCOF-TAEA revealed a consistent pseudoequilibrium CO_2_ capacity (*C*/*C*_0_ = 0.95) of ∼0.47 mmol g^–1^ cycle^–1^ over 3 cycles at 25 °C and 0% RH, ∼1.15 mmol g^–1^ cycle^–1^ over 7 cycles at 25 °C and 70% RH, and ∼1.26 mmol g^–1^ cycle^–1^ over 3 cycles at 15 °C and 70% RH, with no significant decrease in uptake performance, confirming the excellent cycling stability under both ambient and sub-ambient DAC conditions.

**Figure 6:**
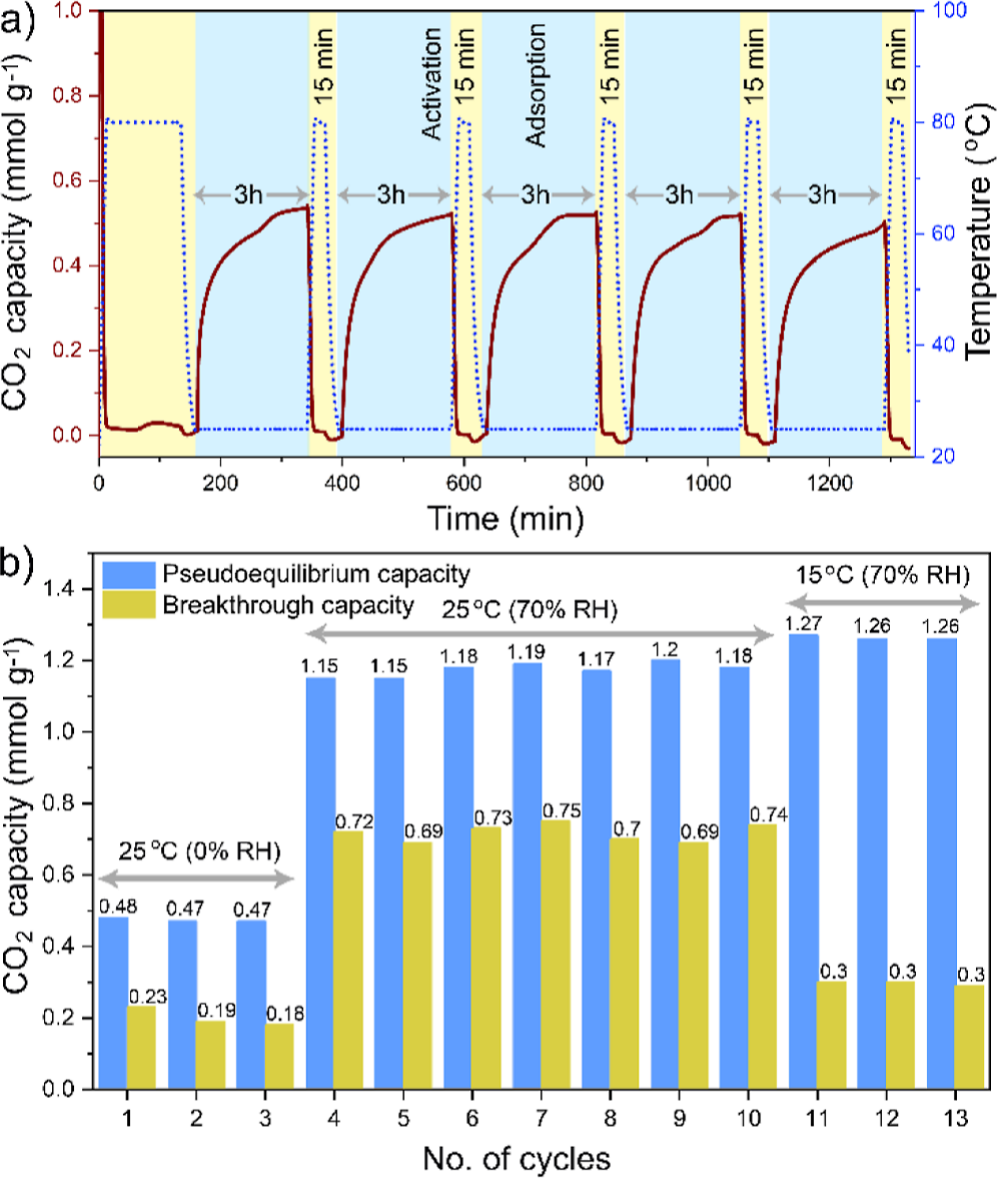
(a) Recyclability of ImCOF-TAEA for CO_2_ capture (400 ppm of CO_2_/N_2_; flow rate: 90 sccm) at 25 °C under dry conditions over 5 consecutive adsorption–desorption cycles, monitored through TGA. Each cycle involved 3 h adsorption followed by 15 min regeneration under N_2_ flow at 80 °C. (b) Pseudoequilibrium (at *C*/*C*_0_ = 0.95) and breakthrough (at *C*/*C*_0_ = 0.05) capacities of ImCOF-TAEA (loading amount: 94.5 mg) measured via fixed bed breakthrough analysis over 13 cycles under three different conditions: 3 cycles under 0% RH at 25 °C, 7 cycles under 70% RH at 25 °C, and 3 cycles under 70% RH at 15 °C (flow rate: 40 sccm 400 ppm of CO_2_/N_2_).

We also observed that the breakthrough capacity (*C*/*C*_0_ = 0.05) at 25 °C increased significantly from ∼0.2 to ∼0.7 mmol g^–1^ cycle^–1^ when switching from dry to humid conditions, highlighting the crucial role of moisture in enhancing CO_2_ capture (Figure 6b). Although the pseudoequilibrium capacities were higher at 15 °C than at 25 °C under humid conditions, the corresponding breakthrough capacity dropped to ∼0.3 mmol g^–1^ cycle^–1^, resulting from slower mass transfer or less efficient CO_2_ chemisorption. Furthermore, the results demonstrated that a relatively low regeneration temperature of 80 °C was sufficient to fully desorb CO_2_ from ImCOF-TAEA. This might be attributed to its low water uptake, which minimizes the energy required for removing adsorbed water during regeneration. No significant change in the FT-IR spectra and FE-SEM images of the fresh and recycled ImCOF-TAEA corroborated the preserved morphology and structural composition after recycling (Figures S26 and S27).

While the present amine-functionalized COFs showed promising DAC performance across a broad temperature range, including sub-ambient and ambient conditions, with excellent recyclability, some aspects still warrant further investigation. First, translating these results to an industrial scale remains challenging due to the expensive precursors and the multistep synthesis routes. Future efforts could focus on developing more cost-effective and scalable synthetic methodologies for amine-functionalized COFs. Second, the recyclability of the materials should be further evaluated using air to better understand their long-term stability toward oxidation. Third, the formation of bicarbonate species under humid conditions could be further confirmed through complementary techniques, such as solid-state ^13^C NMR. Nevertheless, the present study lays a strong foundation for advancing amine-functionalized COFs as efficient adsorbents for DAC under both ambient and sub-ambient conditions across a wide range of humidity.

## Conclusion

In this study, we have explored, for the first time, different amine-grafted COFs for the direct air capture of CO_2_ under wide range of ambient and sub-ambient temperatures from 25 °C to −20 °C and also under varying relative humidity (0% to 70%). Tris­(2-aminoethyl)­amine-functionalized COF, i.e., ImCOF-TAEA, showed comparatively high pseudoequilibrium capacity of 0.46 ± 0.02 mmol g^–1^ at 25 °C under dry conditions. Upon coadsorption in the presence of 70% RH leads to ∼137% increase in the uptake capacity to 1.09 ± 0.09 mmol g^–1^. By lowering the temperature to −5 °C under 70% RH, the capacity was more than 1.0 mmol g^–1^ and at 15 °C, the capacity was the highest, at 1.25 ± 0.02 mmol g^–1^ (presaturation method: 1.52 mmol g^–1^, Table S4). *In situ* DRIFTS suggested preferential formation of ammonium carbamate pairs under humid conditions over the carbamic and ammonium carbamate formation under dry conditions. ImCOF-TAEA showed excellent recyclability both under ambient and sub-ambient conditions with a relatively low regeneration temperature of 80 °C [can be regenerated at lower temperatures (45–65 °C), as confirmed by the TPD study]. This study underscores the untapped potential of amine-functionalized COFs as a varsatile platform for direct air capture of CO_2_ across a wide range of temperatures (25 °C to −20 °C) and humidity conditions (0% to 70% RH). The promising performance of ImCOF-TAEA under both ambient and sub-ambient environments, coupled with its excellent recyclability, and low regeneration temperature, paves the way for further exploration and design of COF-based materials tailored for more geographically flexible direct air capture technologies.

## Experimental Section

### Synthesis of Materials

#### Fabrication of ImCOF

Fabrication of ImCOF was carried out by following a reported procedure with modifications.^39^ 1,3,5-Tris­(4-aminophenyl)­benzene (0.32 mmol) and 2,5-dimethoxyterephthalaldehyde (0.48 mmol) were dispersed in a 4 mL 4:1 (v/v) mixture of mesitylene and 1,4-dioxane in a Schlenk tube and 0.6 mL 6 M acetic acid was then added. Three consecutive freeze–pump–thaw cycles were performed and the reaction mixture was purged with argon, sealed, and heated at 120 °C for 72h. The resulting precipitate was collected by gravimetric filtration, thoroughly washed with methanol and acetone and dried overnight under vacuum at 120 °C, yielding the final ImCOF as a bright yellow powder.

#### Fabrication of ImCOF-Cl

Fabrication of ImCOF-Cl was carried out through post-synthetic modification of ImCOF by following a reported method with minor modifications.^33^ ImCOF (0.61 mmol by imine linkage), anhydrous FeCl_3_ (0.12 mmol), and 2-chloroethyl vinyl ether (9.8 mmol) in diethyl ether (13 mL) were mixed under an inert argon atmosphere. The reaction mixture was gently stirred under 50 °C for 48 h. After cooling to room temperature, the solid was washed with DMF, methanol, and acetone followed by Soxhlet extraction with methanol. The solid was dried overnight under a dynamic vacuum at 120 °C to yield ImCOF-Cl as a yellow solid.

#### Grafting of Polyamines (ImCOF-TAEA, ImCOF-TEPA)

ImCOF-Cl (150 mg) was dispersed in 2.0 mL polyamine [tris­(2-aminoethyl)­amine (TAEA) or tetraethylenepentamine (TEPA)] and two consecutive freeze–pump–thaw cycles were performed followed by argon purging. The reaction was stirred under 120 °C for 24 h. After cooling to room temperature, and the solid washed repetitively with methanol, and acetone. The sample was treated with a 0.5 M sodium hydroxide solution in methanol, followed by ammonium hydroxide solution. The solid collected after centrifugation was thoroughly washed with methanol and acetone. The solid was activated under a dynamic vacuum at 60 °C for 12 h. The product was obtained as dark yellow powder.

#### Characterization

The COF samples were characterized using different spectroscopic and microscopic techniques. DRIFTS measurements were performed on a Thermo Scientific Nicolet iS50 spectrometer. Solid-state ^13^C CP/MAS and HETCOR NMR spectra were recorded on a Bruker AVANCE III 400 MHz instrument equipped with a 9.5 T wide-bore magnet. PXRD data were collected using a Rigaku Miniflex diffractometer with Cu Kα_1_ radiation (λ = 0.15405 nm). Thermal stability was evaluated using a TA Instruments Q500 thermogravimetric analyzer under air. Morphological features were observed using a Hitachi SU8230 FESEM. Textural properties were assessed by N_2_ physisorption isotherms measured on an Autosorb iQ (Anton Paar), while water vapor sorption studies were carried out using a VSTAR analyzer (Anton Paar). CO_2_/N_2_ breakthrough experiments (∼400 ppm of CO_2_) were conducted in a custom-built fixed-bed setup across temperatures ranging from −20 to 25 °C and 0–70% RH. Additional experimental details are provided in the Supporting Information.

## Supplementary Material



## Notes

Spectroscopic and microscopic characterization data of COFs (FTIR, SSNMR, PXRD, TGA, SEM, CHN analysis, and N_2_ physisorption), water vapor adsorption, CO_2_ adsorption behavior and cycling test of amine-grafted COFs (TGA data, and breakthrough data, TPD data) and mechanistic investigation through DRIFTS (PDF)
