# Supplementary material for: Humidity-Enhanced Direct Air Capture of Carbon Dioxide Using Amine-Grafted Covalent Organic Frameworks Under Ambient and Sub-ambient Temperatures
Source: Chem Mater. 2026 Mar 10;38(6):2743–54. doi: 10.1021/acs.chemmater.5c02891 (PMC13019625; doi:10.1021/acs.chemmater.5c02891)
Supplement: Supplementary file 1 [file cm5c02891_si_001.pdf]

# Humidity-Enhanced Direct Air Capture of Carbon Dioxide using Amine-Grafted Covalent Organic Frameworks under Ambient and Sub-Ambient Temperatures

Arkaprabha Giri, Jiaqi Zhang, Xin Deng, and Christopher W. Jones*
School of Chemical & Biomolecular Engineering, Georgia Institute of Technology, Atlanta, GA 30332, USA
E-mail: cjones@chbe.gatech.edu


| Section | Content | Page No. |
|---|---|---|
| **1.** | **Chemicals** | 2 |
| **2.** | **Synthesis of Materials** | 2 |
| 2.1 | Fabrication of ImCOF | 2 |
| 2.2 | Fabrication of ImCOF-Cl | 2 |
| 2.3 | Chemical Grafting of Polyamines (ImCOF-TAEA, ImCOF-TEPA) | 2 |
| **3.** | **Structural Characterizations** | 3-8 |
| 3.1 | Fourier Transformed Infrared (FTIR) Spectroscopic Analysis | 3 |
| 3.2 | Thermogravimetric Analysis (TGA) | 4 |
| 3.3 | Solid-state $^{13}C$-Cross-Polarization/Magic-Angle Spinning (CP/MAS) NMR | 5 |
| 3.4 | Solid-state $^{1}H$-$^{13}C$ Heteronuclear Correlation spectroscopy (HETCOR) NMR | 6 |
| 3.5 | Powder X-ray Diffraction (PXRD) Analysis | 7 |
| 3.6 | Elemental Analysis | 8 |
| 3.7 | Field Emission Scanning Electron Microscopic (FESEM) Imaging | 9 |
| 3.8 | Surface Area and Pore Size Distribution Analyses | 10 |
| **4.** | **Direct Air Capture of $CO_2$** | 12-25 |
| 4.1 | Thermogravimetric Analysis (TGA) | 12 |
| 4.2 | Single Component Vapour Sorption Isotherm Measurements | 13 |
| 4.3 | Fixed Bed Breakthrough Analysis | 15 |
| 4.4 | Temperature Programmed Desorption (TPD) Study | 17 |
| 4.5 | *In Situ* DRIFTS Analysis of $CO_2$ Adsorption under Dry and Humid Conditions | 19 |
| 4.6 | Energy for Dry and Humid $CO_2$ Desorption from ImCOF-TAEA | 21 |
| 4.7 | Summary of the Moisture-Enhanced $CO_2$ Capture Mechanism | 22 |
| 4.8 | Recyclability of ImCOF-TAEA | 24 |
| **5.** | **Comparative Table** | 26 |
| **6.** | **References** | 27 |

## 1. Chemicals

1,3,5-Tris(4-aminophenyl)benzene (purity ≥ 97%) and 2,5-dimethoxyterephthalaldehyde (purity≥ 97%) are obtained from BLD Pharmatech. 2-Chloroethyl vinyl ether (purity > 97%) and tris(2-aminoethyl)amine (purity > 97%) were obtained from TCI Chemicals. Anhydrous $FeCl_3$ (purity >97%), tetraethylenepentamine (purity > 97%), anhydrous diethyl ether, mesitylene, 1,4-dioxane are obtained from Sigma Aldrich. *N,N*-Dimethylformamide (DMF, ACS reagent grade), methanol (ACS reagent grade), and acetone (ACS reagent grade) were obtained from VWR.

## 2. Synthesis of Materials

### 2.1 Fabrication of ImCOF

Fabrication of ImCOF was carried out by following a reported procedure with modifications.[1] 1,3,5-Tris(4-aminophenyl)benzene (112.4 mg, 0.32 mmol) and 2,5-dimethoxyterephthalaldehyde (93.2 mg, 0.48 mmol) were dispersed in a 4 mL 4:1 (v/v) mixture of mesitylene and 1,4-dioxane in a 50 mL Schlenk tube and 0.6 mL 6M acetic acid was then added. Three consecutive freeze-pump-thaw cycles were performed and the reaction mixture was purged with argon, sealed, and heated at 120°C for 72h. The resulting precipitate was collected by gravimetric filtration, thoroughly washed with methanol and acetone, and dried overnight under dynamic vacuum at 120°C, yielding the final ImCOF as a bright yellow powder (yield: ~92%).

### 2.2 Fabrication of ImCOF-Cl

Fabrication of ImCOF-Cl was carried out through post-synthetic modification of ImCOF by following a reported method with minor modifications.[2] ImCOF (120 mg, 0.61 mmol by imine linkage), anhydrous $FeCl_3$ (20 mg, 0.12 mmol), and 2-chloroethyl vinyl ether (1 mL, 9.8 mmol) in diethyl ether (13 mL) were mixed under an inert argon atmosphere, in a 50 mL sealed tube. The reaction mixture was gently stirred under 50°C for 48 h. After cooling to room temperature, the solid was washed with copeus amount of DMF, methanol, and acetone followed by Soxhlet extraction with methanol for 1 day. The solid was dried overnight under a dynamic vacuum at 120°C to yield ImCOF-Cl as a yellow solid (apparent yield: ~90%).

### 2.3 Chemical Grafting of Polyamines (ImCOF-TAEA, ImCOF-TEPA)

ImCOF-Cl (150 mg) was dispersed in 2.0 mL polyamine [tris(2-aminoethyl)amine (TAEA) or tetraethylenepentamine (TEPA)] in a 25 mL Schlenk tube and two consecutive freeze-pump-thaw cycles were performed followed by argon purging. The reaction was stirred under 120°C for 24 h. After cooling to room temperature, and the solid washed repetitively with methanol, and acetone. The sample was placed in a centrifuge tube and shaken using a mechanical shaker with a 0.5 M sodium hydroxide solution in methanol, followed by treatment with ammonium hydroxide solution. The solid collected after centrifugation was thoroughly washed with methanol and acetone. The solid was activated under a dynamic vacuum at 60°C for 12 h. The product was obtained as dark yellow powder.

**N.B.:** We avoided post-synthetic treatments that could increase the carbon footprint, such as supercritical $CO_2$ drying or freeze-drying.

## 3. Structural Characterizations

### 3.1 Fourier Transformed Infrared (FTIR) Spectroscopic Analysis

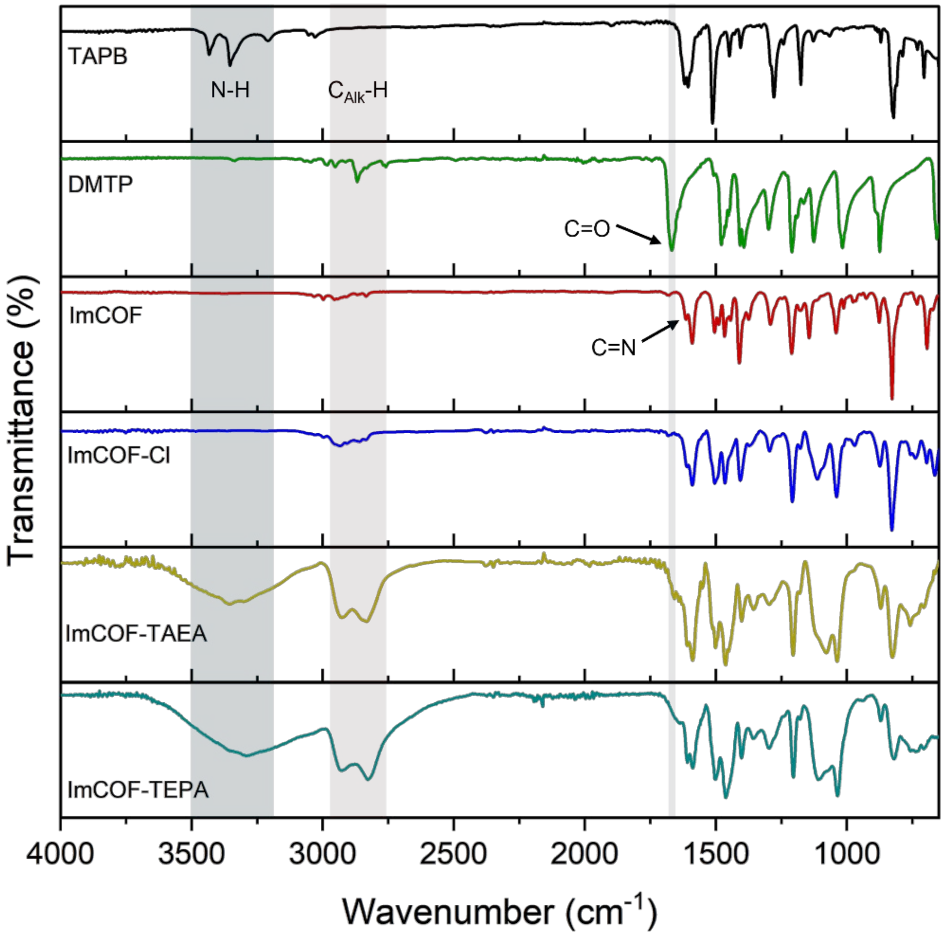


**Figure S1.** FTIR spectroscopic analysis of ImCOF, ImCOF-Cl, ImCOF-TAEA, ImCFO-TEPA, and constituent monomers of ImCOF, such as 1,3,5-tris(4-aminophenyl)benzene (TAPB) and 2,5-dimethoxyterephthalaldehyde (DMTP). The measurements were carried out using Thermo Fisher Scientific Nicolet 6700 FT-IR instrument in attenuated total reflection (ATR) mode with 64 scan and spectral resolutions of 4 cm$^{-1}$.

### 3.2 Thermogravimetric Analysis (TGA)

The differential TGA curves for the pristine and amine-grafted COFs under air showed two distinct decomposition domains. The first mass-loss begins at around 150-200°C, corresponding to the thermal degradation of the grafted amines (i.e., TAEA, TEPA) and the 2-chloroethoxy groups. The second, sharper mass-loss begins at ~380°C, which is attributed to the decomposition of the framework functional groups (such as imine, methoxy, and tetrahydroquinoline linkages), followed by degradation of the aromatic COF backbone from ~450°C onwards.

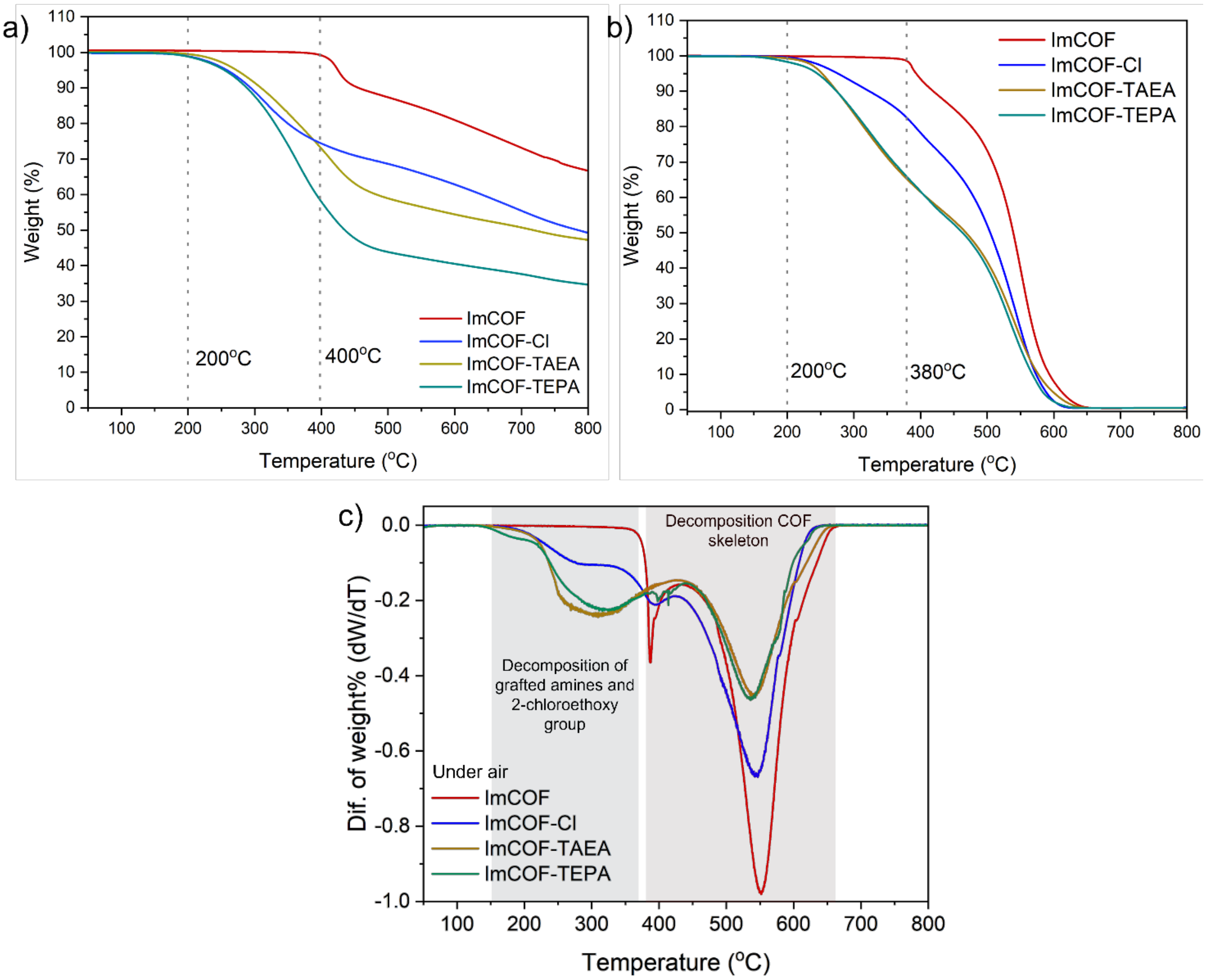


**Figure S2.** TGA of ImCOF, ImCOF-Cl, ImCOF-TAEA, and ImCOF-TEPA under (a) $N_2$ and (b) air. (c) Differential TGA plot of ImCOF, ImCOF-Cl, ImCOF-TAEA, and ImCOF-TEPA under air. The analyses were carried out using TGA 550 instrument.

### 3.3 Solid-state $^{13}C$-Cross-Polarization/Magic-Angle Spinning (CP/MAS) NMR

$^{13}C$ CP/MAS NMR was conducted on the Bruker Avance III (400 MHz) with a 9.5 T wide-bore magnet. Dry powder samples were packed tightly in a 4 mm zirconia rotor. Adamantane and glycine were used as references prior to sample analysis. The MAS rate was controlled at 12 kHz.

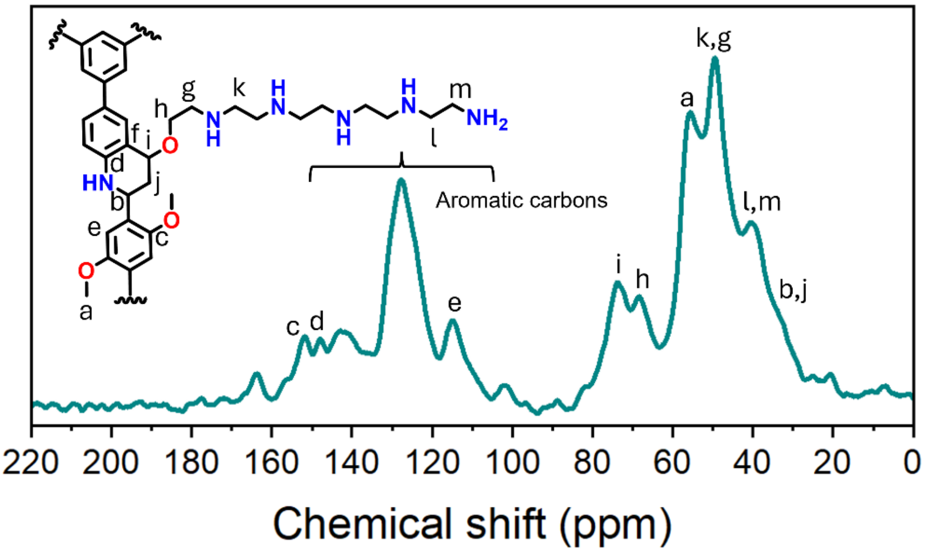


**Figure S3.** Solid-state $^{13}C$ CP/MAS NMR spectra of ImCOF-TEPA.

### 3.4 Solid-state $^{1}H$-$^{13}C$ Heteronuclear Correlation spectroscopy (HETCOR) NMR

$^{1}H$-$^{13}C$ HETCOR NMR was conducted on the Bruker Avance III (400 MHz) with a 9.5 T wide-bore magnet. Dry powder samples were packed tightly in a 4 mm zirconia rotor. Adamantane and glycine were used as references prior to sample analysis. The MAS rate was controlled at 12 kHz. The contact time was fixed at 2000 μs. The 90° pulse widths were 1.0 and 6.3 μs in the $^{1}H$ and $^{13}C$ channels, respectively.

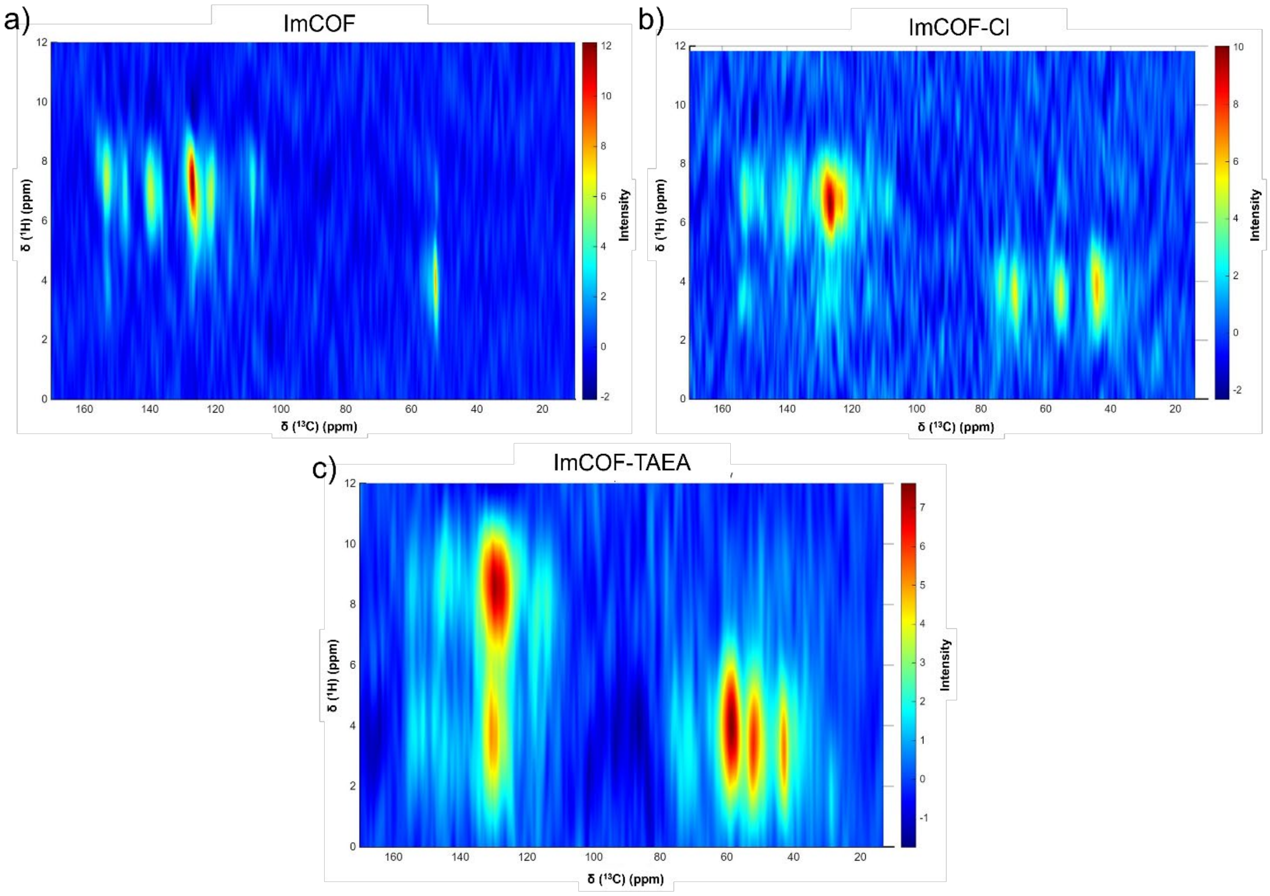


**Figure S4.** $^{1}H$-$^{13}C$ HETCOR spectra of (a) ImCOF, (b) ImCOF-Cl, and (c) ImCOF-TAEA.

## 3.5 Powder X-ray Diffraction (PXRD) Analysis

PXRD measurements were performed on a Rigaku Miniflex diffractometer using Cu K$\alpha_1$ radiation ($\lambda$ = 0.15405 nm). COF powder was mounted on a silicon zero-background holder, spread evenly, and flattened with a glass plate. Data were collected over the 2θ range of 2-40° at a scan rate of 10° min$^{-1}$. Pawley refinement of the experimental PXRD of ImCOF was carried out using BIOVIA Materials Studio (version 6.1), and the refined unit cell parameters were consistent with previous literature report (Figure S5a).[1] Pawley refinement of ImCOF-Cl (considering 100% tetrahydroquinoline linkage) gave unit cell parameters: a = 37.36 Å, b = 36.99 Å, c = 4.96 Å; α = β = 90°, γ = 120° (Figure S5b).

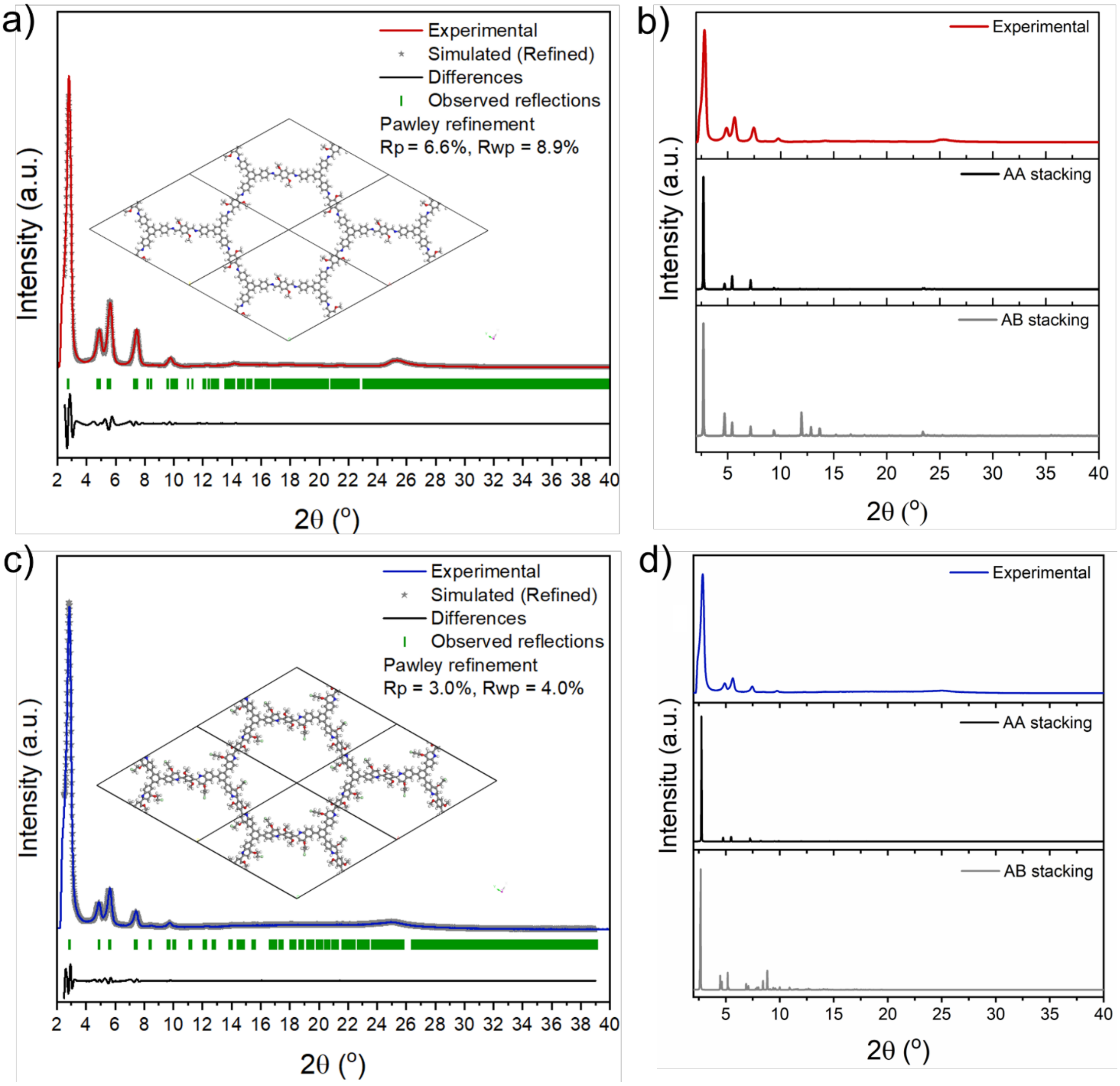


**Figure S5.** Experimental PXRD (red/blue), Pawley refined profile (grey star), observed reflections (green), differences between refined and experimental profile (black) for (a) ImCOF (inset: optimized ImCOF structure in the unit cell) and (c) ImCOF-Cl (inset: optimized ImCOF-Cl structure in the unit cell). Comparison between experimental PXRD pattern with the simulated AA and AB stacking mode of (b) ImCOF, (c) ImCOF-Cl.

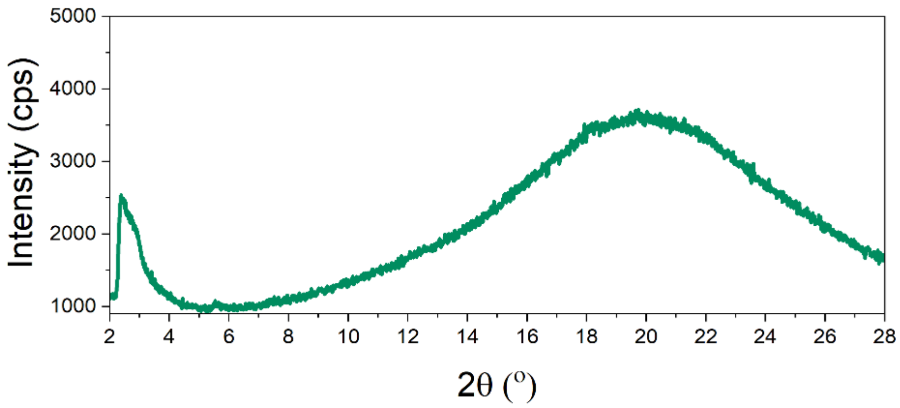


**Figure S6.** Experimental PXRD pattern of ImCOF-TEPA. The random orientation of TEPA within the COF pores results in weakened diffraction peaks.[3]

### 3.6 Elemental Analysis

**Table S1.** Summary of the elemental (C, H, N, Cl) analyses# of various COF samples.

| Elements (wt.%) | ImCOF | | ImCOF-Cl | | ImCOF-TAEA | | ImCOF-TEPA | |
|---|---|---|---|---|---|---|---|---|
| | Theo. | Expt. | Theo. | Expt. | Theo. | Expt.[1] | Theo. | Expt.[2] |
| C | 79.6 | 78.9 | 67.4 | 68.3 | 67.0 | 63.8 | 65.9 | 64.8 |
| H | 5.1 | 5.1 | 5.7 | 6.0 | 8.3 | 8.0 | 8.6 | 7.8 |
| N | 7.1 | 7.2 | 4.6 | 4.1 | 17.0 | 13.2 | 18.5 | 13.1 |
| O$ | 8.2 | - | 10.6 | - | 7.8 | - | 7.0 | - |
| Cl | - | - | 11.7 | 8.4 | 0 | 0 | - | - |

[1]Nitrogen content of three different batches of ImCOF-TAEA (13.2, 13.0, 12.5 wt.%)
[2]Nitrogen content of two different batches of ImCOF-TEPA (13.1, 13.7 wt.%)
$Oxygen in the sample does not generate a distinct analyzable gas during combustion and cannot be distinguished from the excess oxygen supplied for the reaction. Therefore, CHN elemental analysis cannot provide reliable quantitative oxygen content.
#The elemental analyses of the COF samples were conducted at Atlantic Microlab, Inc.

### 3.7 Field Emission Scanning Electron Microscopic (FESEM) Imaging

The morphologies of the COF samples were investigated using a Hitachi SU8230 FESEM with accelerating voltage of 5-10 kV. The samples were prepared by sprinkling COF powders on a carbon tape attached with an aluminium stub. The samples were coated with a thin layer (~15 nm) of sputtered gold-palladium and then subjected to imaging. The elemental mapping of the COF samples was investigated through energy-dispersive spectroscopy (EDS) using a Hitachi SU8230 FESEM with accelerating voltage of 20 kV.

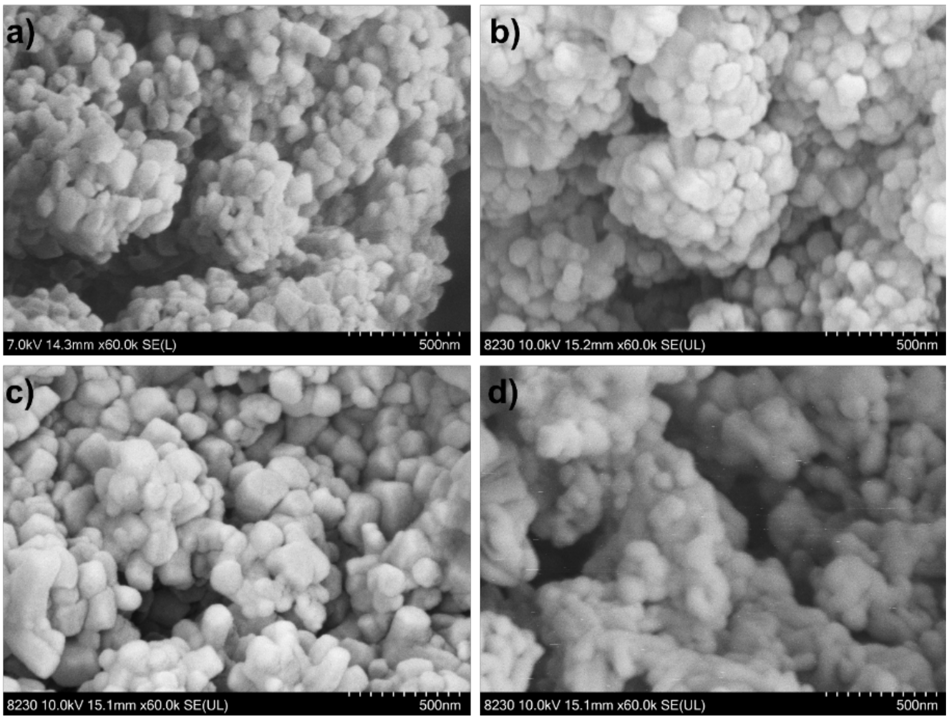


**Figure S7.** FESEM imaging of (a) ImCOF, (b) ImCOF-Cl, (c) ImCOF-TAEA, and (d) ImCOF-TEPA (scale bar = 500 nm).

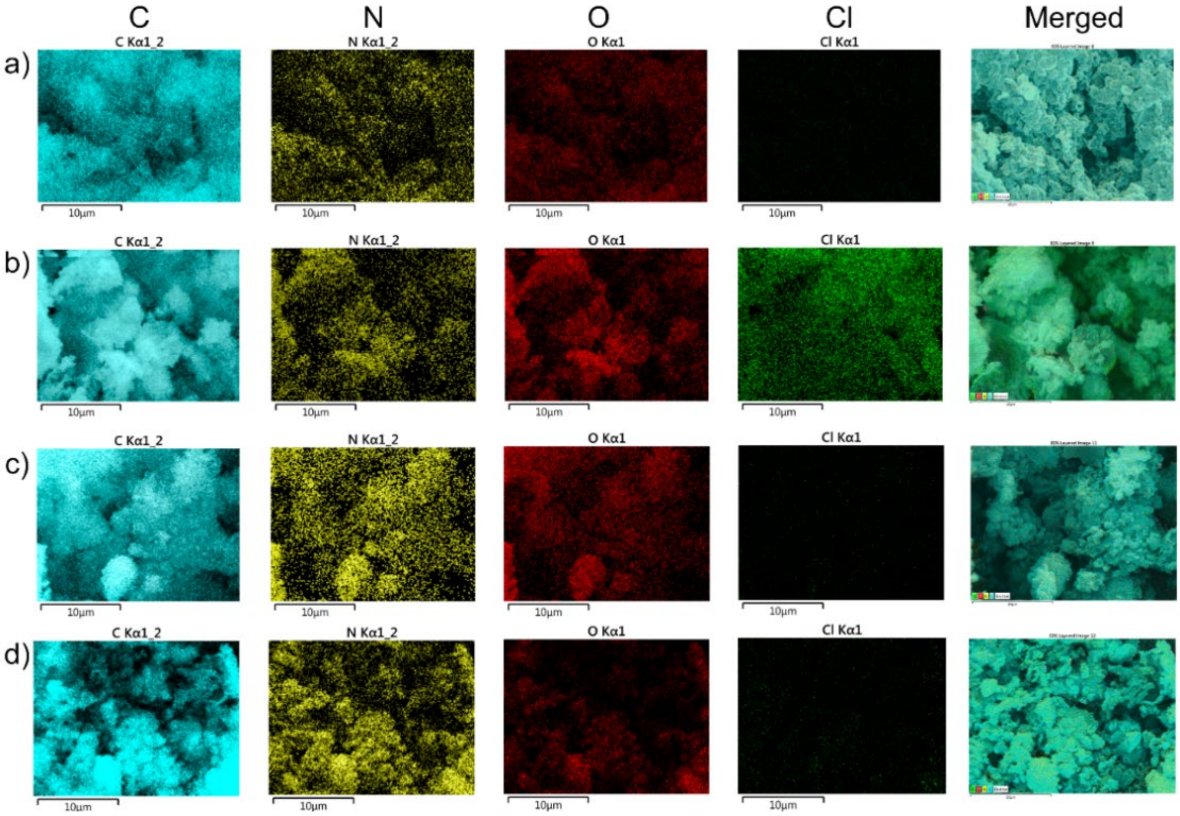


**Figure S8.** Elemental mapping using FESEM of (a) ImCOF, (b) ImCOF-Cl, (c) ImCOF-TAEA, and (d) ImCOF-TEPA (scale bar = 10 μm).

### 3.8 Surface Area and Pore Size Distribution Analyses

$N_2$ physisorption experiments were conducted using Autosorb iQ/Anton Paar instrument. Before the $N_2$ physisorption, COF samples were activated at 120°C for 6 h under vacuum. BET surface area was estimated using the $N_2$ physisorption data in the $P/P_0$ range of 0.05-0.3. Pore volumes of the materials were determined based on the $N_2$ physisorption at a partial pressure of 0.95. The pore size distribution (PSD) was analysed using the quenched solid density functional theory (QSDFT) model, which provides an accurate description of adsorption behaviour in narrow mesoporous materials (0.5-35 nm) by considering for surface roughness and heterogeneity effects.

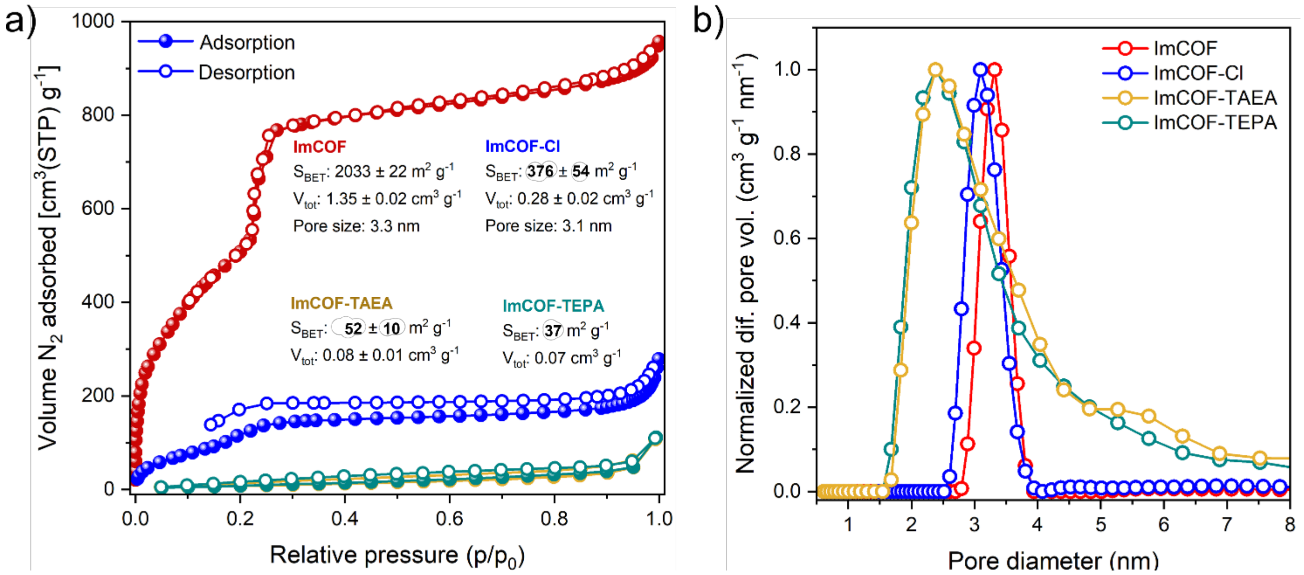


**Figure S9.** (a) Nitrogen physisorption isotherms at 77 K and (b) Quenched solid density functional theory (QSDFT) pore size distribution of ImCOF, ImCOF-Cl, ImCOF-TAEA, and ImCOF-TEPA (carbon, cylindrical pore, adsorption branch model).

The PSD results for amine-grafted COFs are corroborated with Barrett-Joyner-Halenda (BJH) model. To rigorously assess the presence of ultramicropores (< 0.7 nm), we additionally performed $CO_2$ adsorption of ImCOF-TAEA at 273 K and analyzed the data using the non-local density functional theory (NLDFT) model. This analysis revealed that ImCOF-TAEA exhibits a dominant pore at ~0.35 nm, which we attribute to the interlayer spacing between the successive 2D COF sheets, consistent with the expected π–π stacking distance. A weaker feature centered around ~0.69 nm likely arises from defect-induced microporosity. A similar ultramicropore feature (~0.35 nm) was also observed for ImCOF-TEPA.

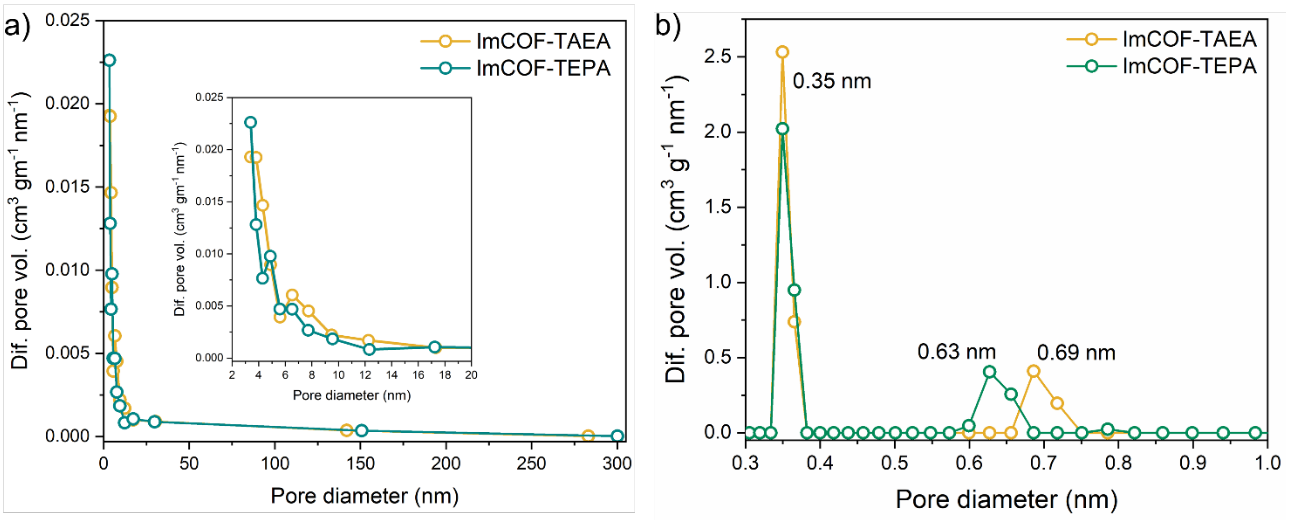


**Figure S10.** (a) The BJH pore size distributions of ImCOF-TAEA and ImCOF-TEPA revealed that, after post-synthetic amine grafting, the pores remained concentrated in the narrow mesoporous region (dominant peak at ~3.5 nm), indicating the preservation of the 1D pore channels of COF. (b) NLDFT pore size distribution of ImCOF-TAEA and ImCOF-TEPA using $CO_2$ adsorption analysis.

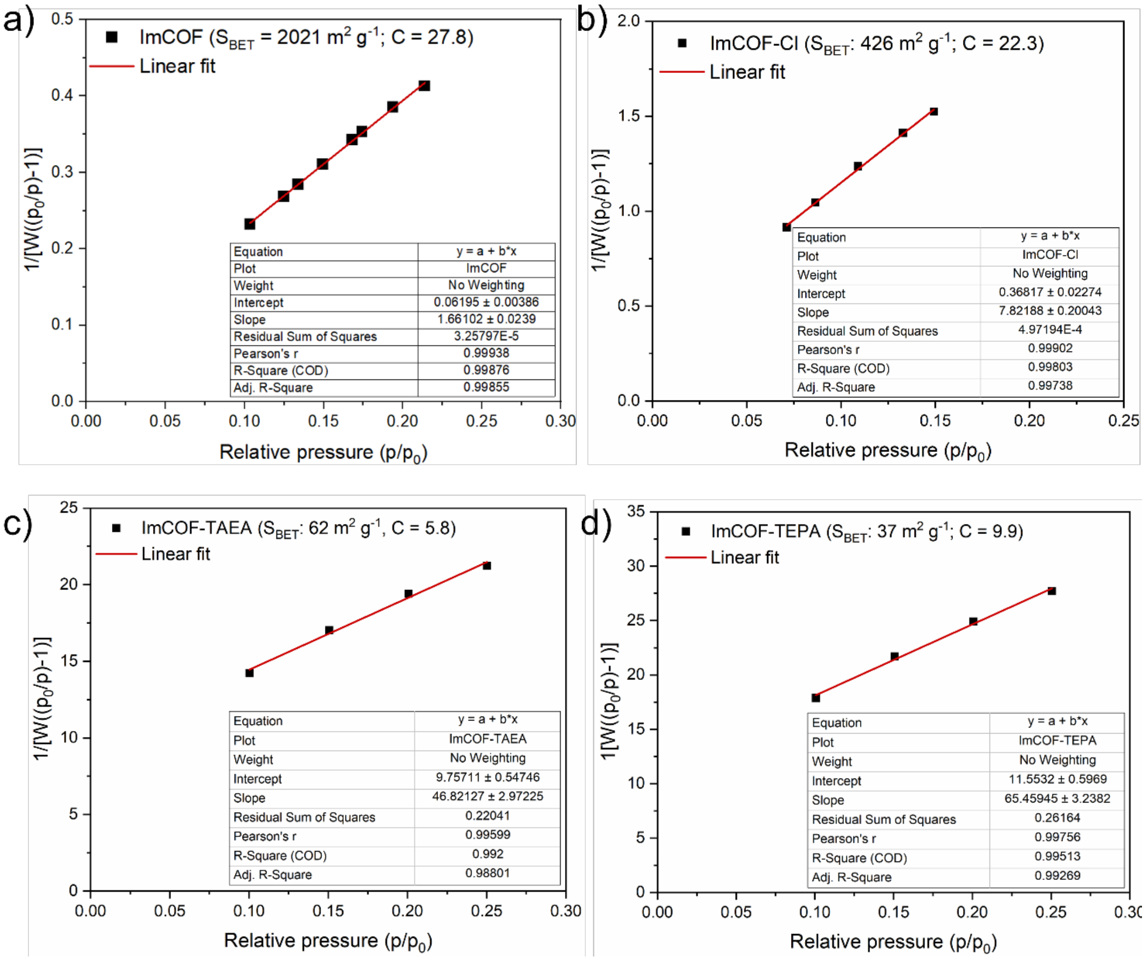


**Figure S11.** Specific BET surface area plots of (a) ImCOF, (b) ImCOF-Cl, (c) ImCOF-TAEA, and (d) ImCOF-TEPA.

**Table S2.** Summary of the specific BET surface area, total pore volume and pore size of various COF samples

| COFs | $S_{BET}$ ($m^2/g$) of different batches | $S_{BET}$ ($m^2/g$) | $V_{tot}$ ($cm^3/g$) at $p/p_0 = 0.95$ | Pore size (nm) |
|---|---|---|---|---|
| ImCOF | 2021, 2014, 2064 | 2033 ± 22 | 1.35 ± 0.02 | 3.3 |
| ImCOF-Cl | 426, 287, 381, 408 | 376 ± 54 | 0.28 ± 0.02 | 3.1 |
| ImCOF-TAEA | 62, 42 | 52 ± 10 | 0.08 ± 0.01 | 2.4 |
| ImCOF-TEPA | 37 | 37 | 0.07 | 2.4 |

## 4. Direct Air Capture of $CO_2$

### 4.1 Thermogravimetric Analysis (TGA)

TGA measurements were carried out in TA Instrument Q500. The COF samples (~10 mg) were placed in a 50 µL platinum pan, heated to 120°C at a ramp rate of 10°C $min^{-1}$, held at 120°C for 3 h under a 90 sccm $N_2$ flow, and then cooled down to 30°C at a rate of 10°C $min^{-1}$. Then exposed to the respective $CO_2/N_2$ mixture (90 sccm) for 12 h.

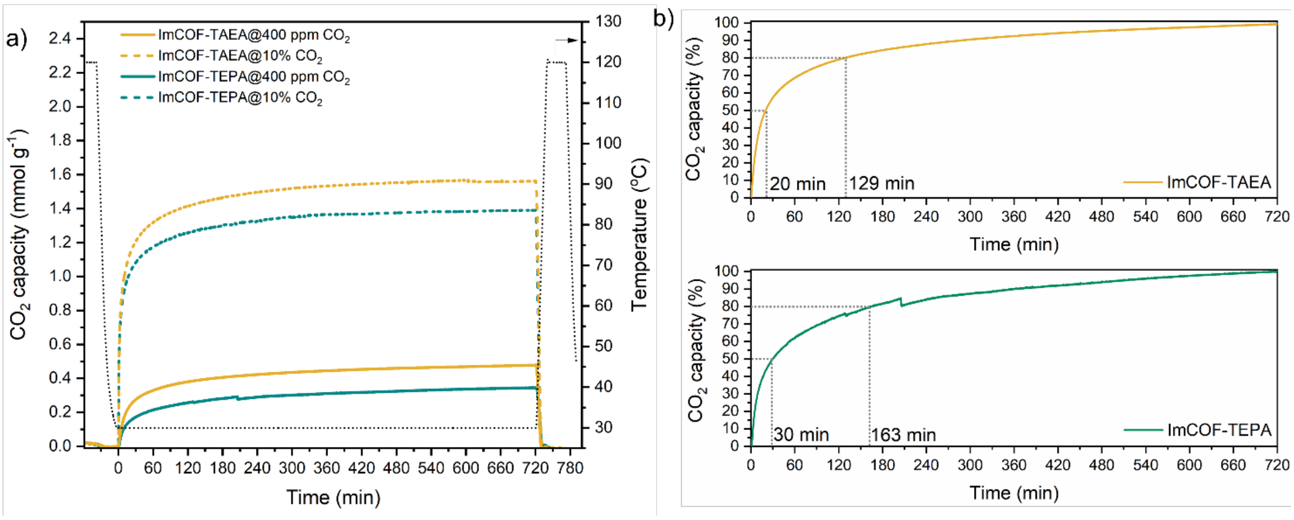


**Figure S12.** (a) Thermogravimetric analysis (TGA) of $CO_2$ adsorption on ImCOF-TAEA (yellow), and ImCOF-TEPA (cyan), under 400 ppm $CO_2/N_2$ (bold line) and 10% $CO_2/N_2$ (dotted line) at 30°C and 0% relative humidity. The corresponding temperature profile is shown as black dotted lines. (b) Time (min) vs. % of total $CO_2$ uptake capacity for ImCOF-TAEA and ImCOF-TEPA under dry DAC (400 ppm $CO_2/N_2$) conditions at 30 °C.

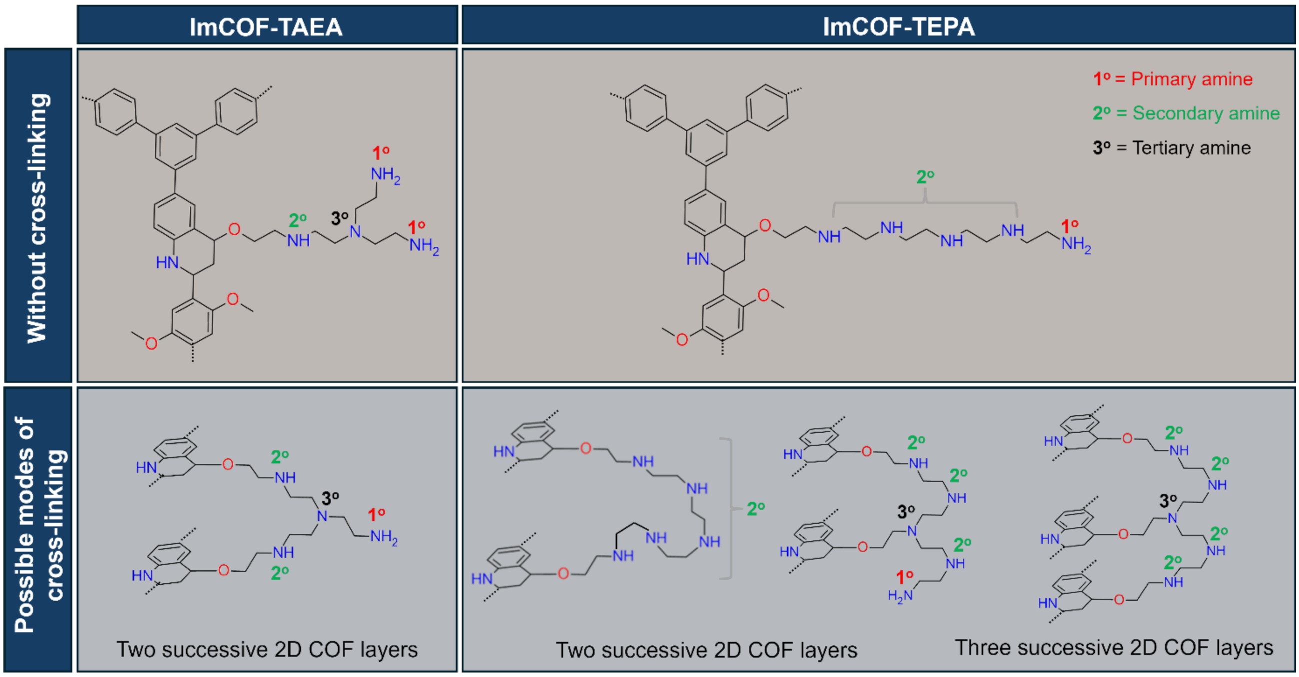


**Figure S13.** Pictorial illustration highlighting the distribution of primary, secondary, and tertiary amines in ImCOF-TAEA and ImCOF-TEPA, presented for both crosslinked and non-crosslinked states.

### 4.2 Single Component Vapor Sorption Isotherm Measurements

Water vapor sorption experiments were conducted using a VSTAR instrument (from Anton Paar). Powder samples (30-50 mg) were degassed under dynamic vacuum at 120°C for 6 hours. Ultra-high-purity helium was used for free space corrections, while an isothermal bath maintained the sample temperature throughout the measurements. The isosteric heat of water vapour adsorption was estimated using Clausius-Clapeyron equation based on water vapour sorption isotherms at 25°C and 5°C and plotted as a function of uptake. ImCOF shows the lowest water uptake up to 70% RH due to its hydrophobic pore environment. A sharp increase in uptake appears only near ~0.7 $P/P_{sat}$, consistent with capillary condensation in its relatively larger pores. Upon introducing the 2-chloroethoxy tetrahydroquinoline groups, ImCOF-Cl becomes more polar but pore volume decreases. This leads to slightly higher water uptake at lower RH (<70%), driven by increased hydrophilicity, while the total uptake remains lower than that of ImCOF because capillary condensation occurs in smaller pores with lower pore volume. Further incorporation of amine functionalities increases pore polarity even more, enhancing water adsorption at low and intermediate RH. However, the simultaneous reduction in pore volume and pore size limits the overall water uptake capacity at high RH, despite the stronger hydrophilic interactions.

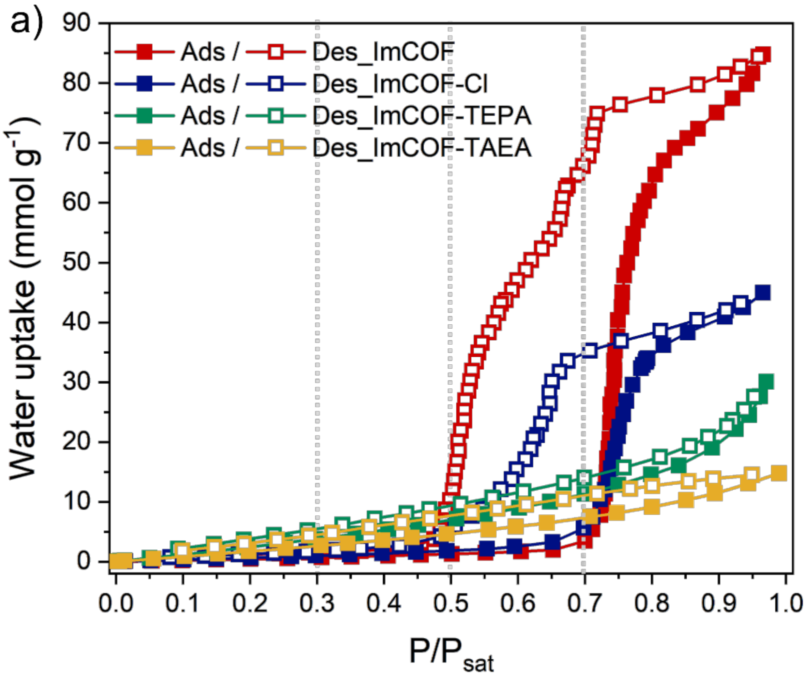


b)

| COF | Water uptake (mmol g$^{-1}$) at 30% RH | at 50% RH | at 70% RH |
|---|---|---|---|
| ImCOF | 0.3 | 1.3 | 3.5 |
| ImCOF-Cl | 1.0 | 1.8 | 5.6 |
| ImCOF-TAEA | 2.6 | 4.7 | 7.3 |
| ImCOF-TEPA | 3.8 | 7.0 | 11.2 |

**Figure S14.** (a) Water vapor sorption isotherms of ImCOF, ImCOF-Cl, ImCOF-TEPA, and ImCOF-TAEA at 25 °C. (b) Summary of water uptake capacities for the COF materials at various relative humidities at 25 °C.

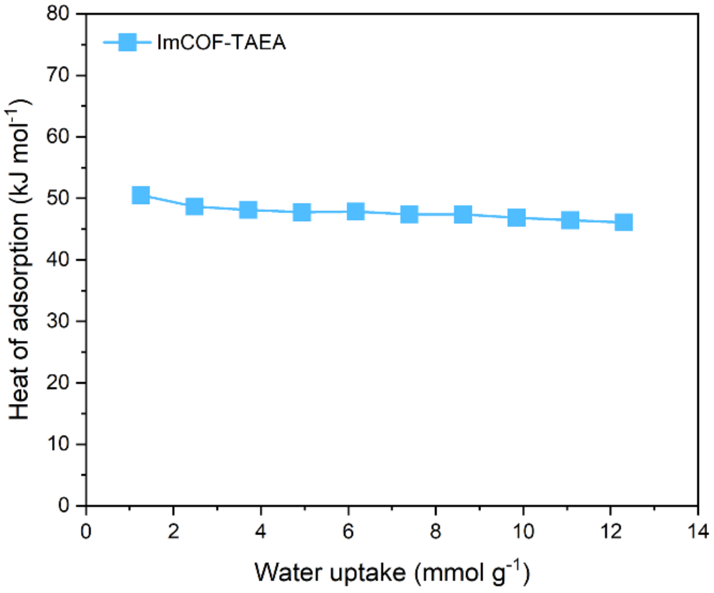


**Figure S15.** The isosteric heat of water vapor adsorption for ImCOF-TAEA.

### 4.3 Fixed Bed Breakthrough Analysis

All the breakthrough experiments with 400 ppm $CO_2/N_2$ under a wide range of temperatures (-20°C to 25°C) and relative humidities (0%-70%) were conducted in a custom-built fixed-bed reactor, as described in detail in our previous report.[4] In brief, activated ImCOF-amine samples (40-100 mg) were packed into a 1/4″ stainless-steel tube with ~5 cm length and 4 mm of internal diameter, with both ends sealed with glass wool (10-15 mg). A coiled 1/8″ stainless-steel tube was installed upstream of the breakthrough fixed bed for controlled pre-heating or pre-cooling of the gas feed before contact with the sorbent. The reactor bed was immersed in a refrigerated circulating liquid bath [1:1 (v/v) water:ethylene glycol] that can maintain constant temperature from -20 to 80°C with programmable ramping rate during experiments, using a Julabo CD-600F chiller. The flow rate of dry $CO_2/N_2$ gases was accurately controlled by Alicat Scientific mass flow controllers. Prior to testing, the samples were activated at 80°C under constant $N_2$ flow (60 sccm) for 2 h. Breakthrough experiments were then performed using dry or humid (0%, 30%, 50%, and 70% RH) 400 ppm $CO_2/N_2$ gas streams at a flow rate of 40 sccm. The humidity levels were precisely controlled by WETSYS/SETARAM humidifier. The outlet $CO_2/H_2O$ concentration was continuously monitored with an infrared gas analyzer, LI-850/LI-COR.

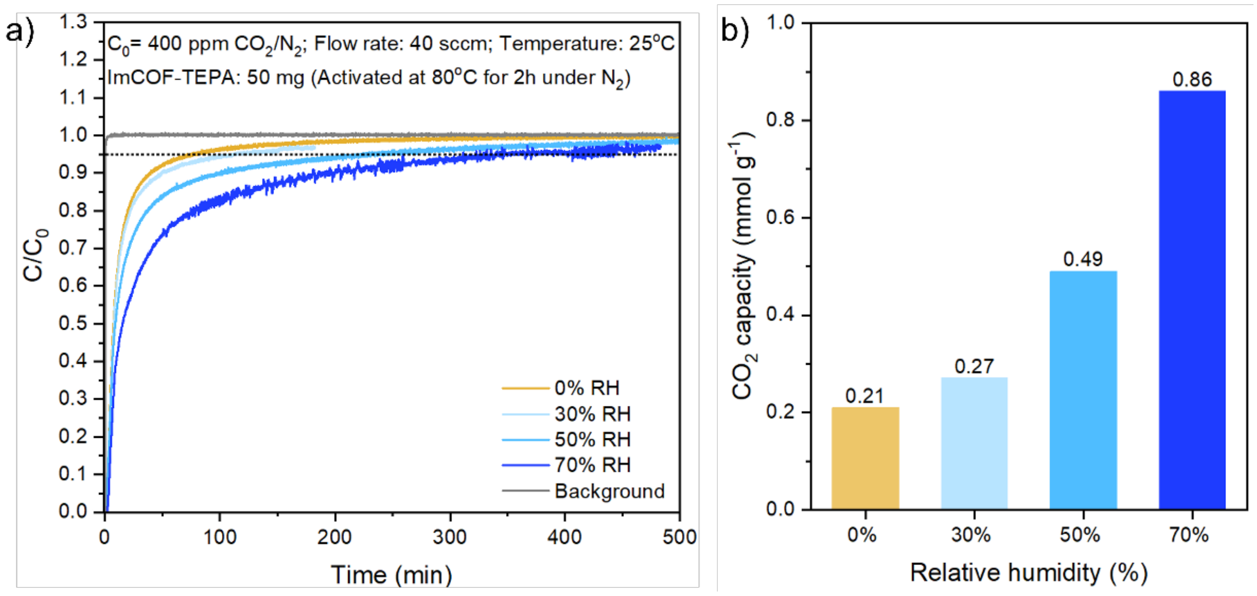


**Figure S16.** (a) $CO_2$ dynamic breakthrough curves and (b) estimated $CO_2$ uptake under 400 ppm of $CO_2/N_2$ with 0%, 30%, 50%, and 70% relative humidity (RH, co-adsorption) at 25°C for ImCOF-TEPA.

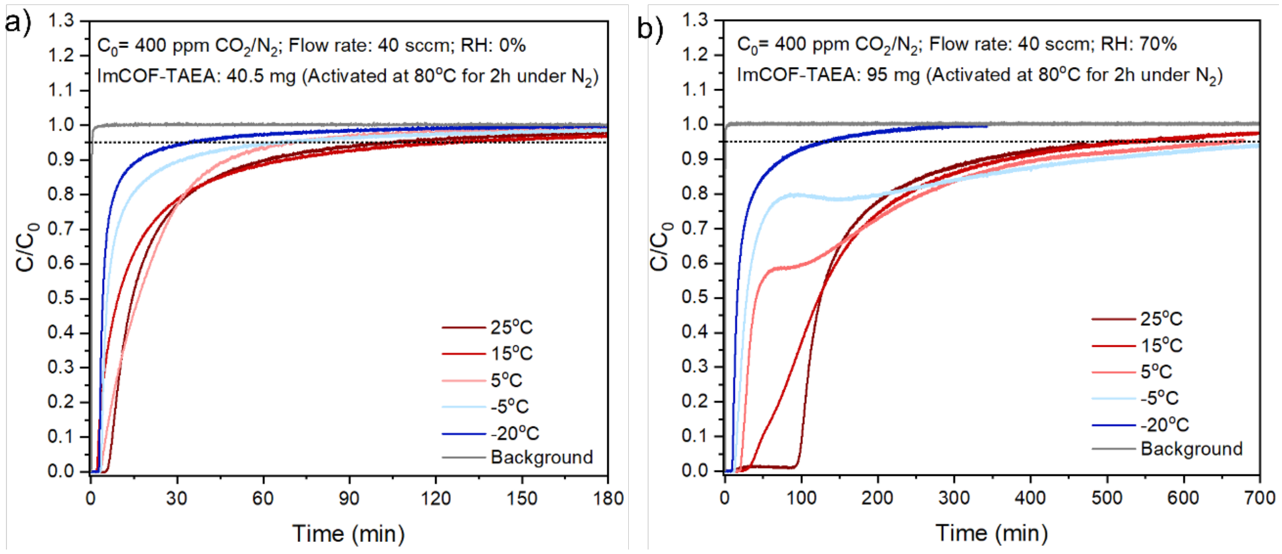


**Figure S17.** $CO_2$ dynamic breakthrough curves under 400 ppm of $CO_2/N_2$ at different temperatures with (a) dry (b) 70% RH conditions (co-adsorption) for ImCOF-TAEA. The slightly deviating breakthrough behavior observed at 5°C is attributed to a kinetic-thermodynamic crossover regime, where enhanced $CO_2$-amine interactions are counterbalanced by reduced diffusion and amine mobility. Under humid conditions, additional competitive adsorption of water further modulates $CO_2$ transport and reaction kinetics, resulting in a non-ideal breakthrough profile.

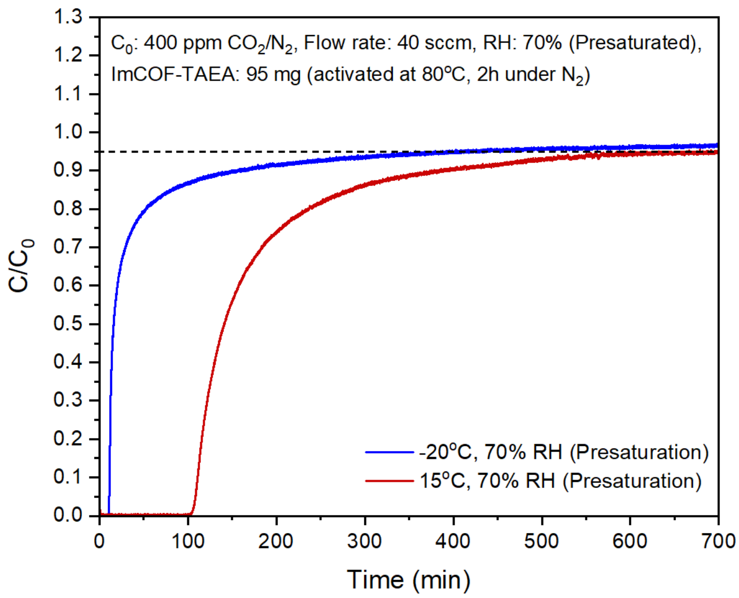


**Figure S18.** $CO_2$ dynamic breakthrough curves under 400 ppm of $CO_2/N_2$ at different temperatures with 70% RH under presaturation condition for ImCOF-TAEA. In case of the presaturation humid experiments, ImCOF-TAEA was pre-humidified with 70% RH/$N_2$ (flow rate: 40 sccm), at the respective temperatures (-20°C, 15°C) before switching to humid 400 ppm $CO_2/N_2$ adsorption (flow rate: 40 sccm).

### 4.4 Temperature Programmed Desorption (TPD) Study

TPD experiments were performed immediately after dry or humid $CO_2$ breakthrough runs (Figure S17-S19). Upon reaching pseudoequilibrium ($C/C_0$ = 0.95), the inlet 400 ppm $CO_2/N_2$ (40 sccm) was switched to pure $N_2$ (60 sccm) and the fixed bed was purged for 45 min at the adsorption temperature (-20, -5, 5, 15, or 25°C). The temperature was then ramped to 80°C at controlled heating rates (0.3 to 0.7°C $min^{-1}$). Outlet $CO_2$ and $H_2O$ concentrations were continuously recorded using a LI-850/LI-COR analyzer to track desorption profiles.

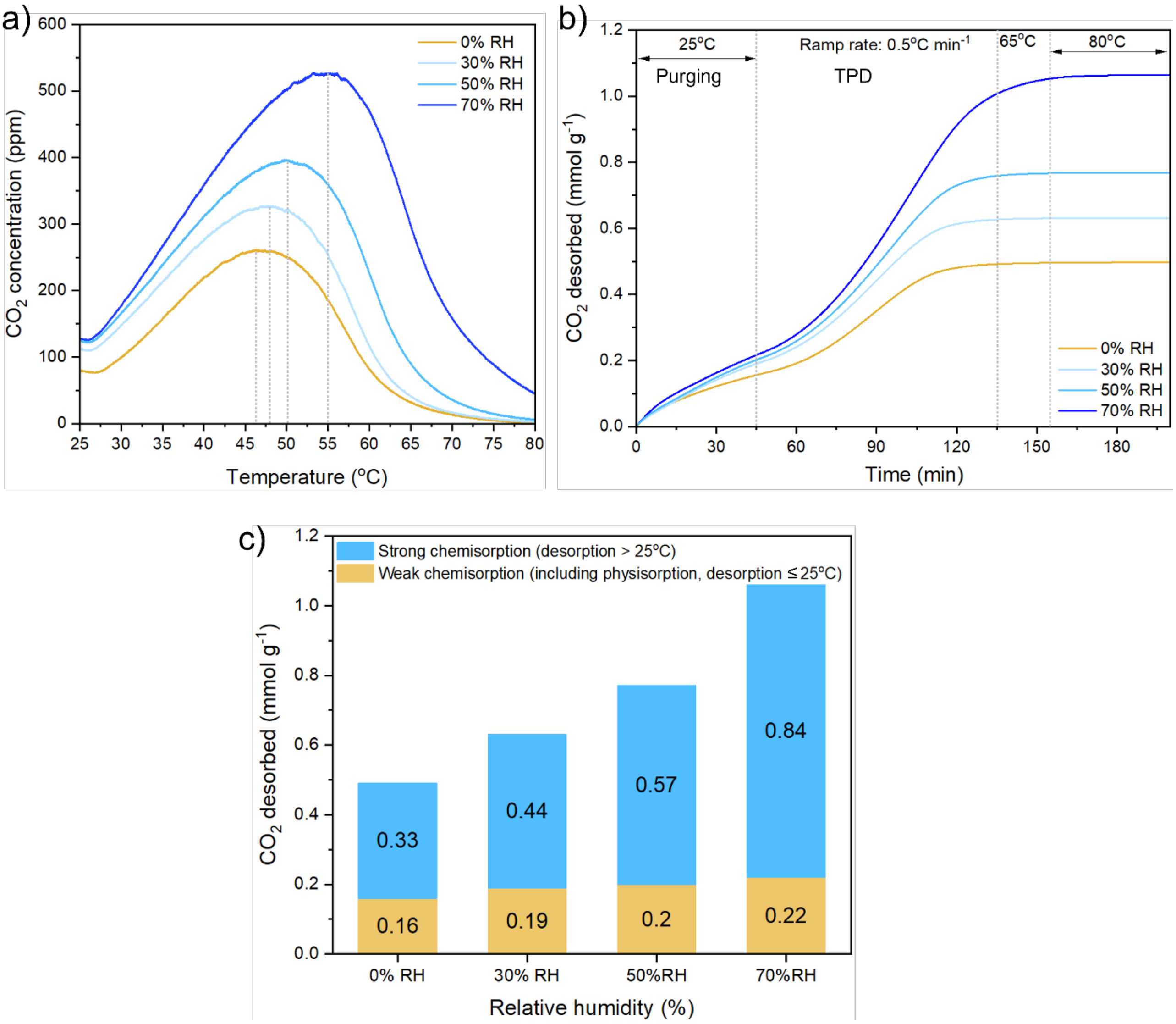


**Figure S19.** Quantified $CO_2$ desorption profiles from TPD experiments (ramp rate: 0.5°C $min^{-1}$) at 25 °C under different relative humidity using ImCOF-TAEA: (a) $CO_2$ concentration (ppm) vs. Temperature (°C) plot and (b) $CO_2$ desorbed (mmol $g^{-1}$) vs. Time (min) plot, (c) Contribution of weak chemisorption (including physisorption) and strong chemisorption in the total amount of desorbed dry/humid $CO_2$ from ImCOF-TAEA during the TPD.

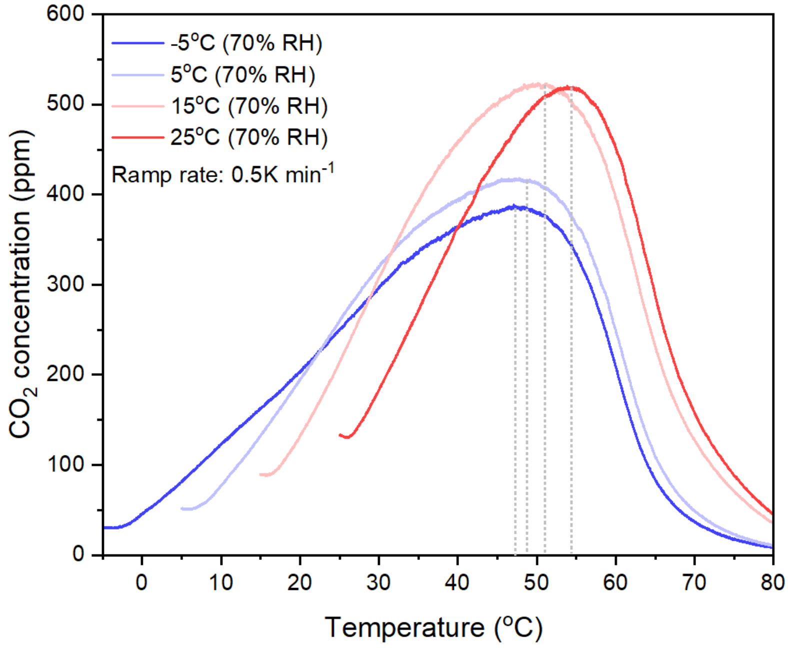


**Figure S20.** TPD experiments of $CO_2$ on ImCOF-TAEA at -5, 5, 15, and 25 °C under 70% relative humidity. Adsorption was performed with 400 ppm $CO_2/N_2$ at a flow rate of 40 sccm. Following adsorption, samples were purged with $N_2$ (60 sccm) at the respective temperature for 45 min, then heated at a ramp rate of 0.5K $min^{-1}$ to 80 °C under 60 sccm $N_2$ flow for 2 h.

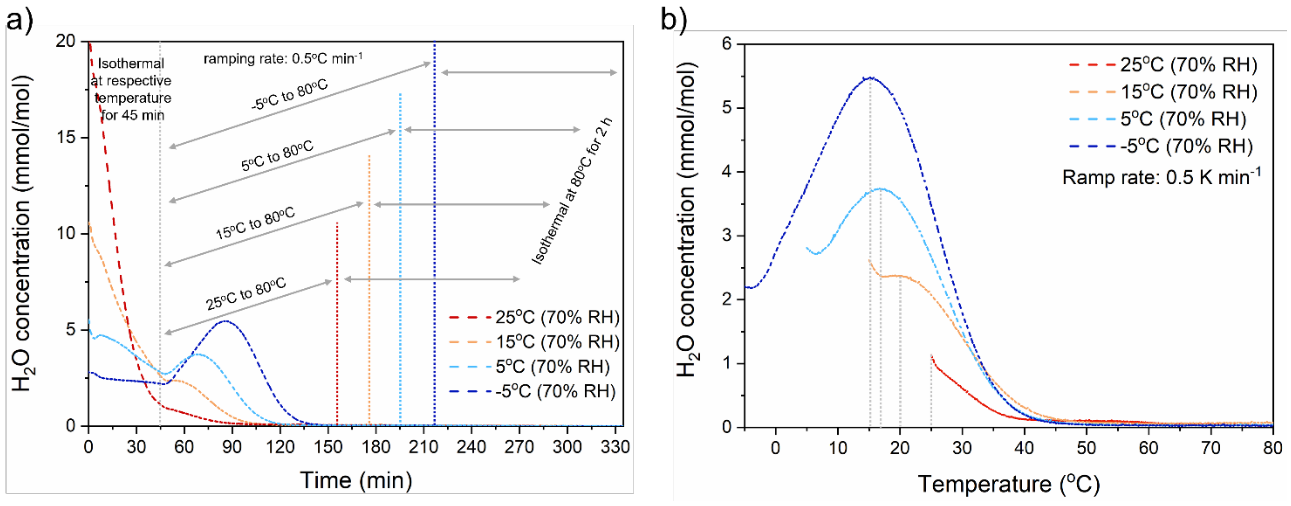


**Figure S21.** TPD experiments of (a) $H_2O$ concentration (mmol/mol) vs time (min) and (b) $H_2O$ on ImCOF-TAEA at -5, 5, 15, and 25 °C under 70% relative humidity. Adsorption was performed with humid 400 ppm $CO_2/N_2$ at a flow rate of 40 sccm. Following adsorption, samples were purged with $N_2$ (60 sccm) at the respective temperature for 45 min, then heated at a ramp rate of 0.5K $min^{-1}$ to 80 °C under 60 sccm $N_2$ flow for 2 h.

### 4.5 *In Situ* DRIFTS Analysis of $CO_2$ Adsorption under Dry and Humid Conditions

*In situ* DRIFTS analyses of amine-grafted COFs under different DAC conditions were performed on a Nicolet iS10 spectrometer. About 50 mg of sample was activated inside the DRIFTS sample holder at 120°C under $N_2$ flow of 60 sccm for 2 h. Then, after lowering the temperature to 25°C and equilibrating for 10 min, the sample was exposed to dry or humid 400 ppm $CO_2/N_2$ with a flow rate of 40 sccm. For humid experiments, 400 ppm $CO_2/N_2$ was pre-humidified by passing it through a saturated aqueous sodium chloride solution using a bubbler at 23°C, providing ~75% relative humidity (RH). IR spectra were collected at fixed intervals (64 scans, 4 $cm^{-1}$ resolution). To better visualize the signals arising from $CO_2$ uptake, the IR spectra collected after exposure to 400 ppm dry/humid $CO_2$ were corrected by subtracting the spectrum of the activated sorbent. The assigned peaks, together with relevant literature references, are listed in Table S3.

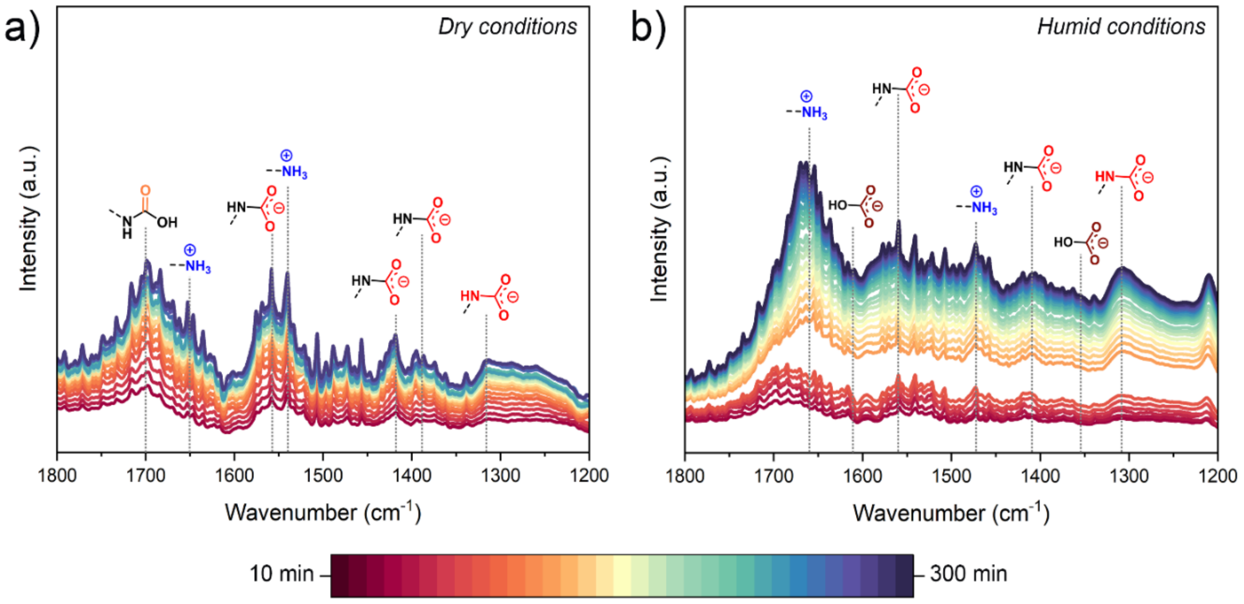


**Figure S22.** *In situ* DRIFT spectra of ImCOF-TEPA as a function of adsorption time at 25°C under (a) dry and (b) humid conditions. Yellow labels: carbamic acid, red labels: carbamate ion, blue labels: ammonium ion.

**Table S3.** Summary table of the DRIFTS peak assignment for the species generated during $CO_2$ sorption by amine functionalized sorbents with corresponding references

| Wavenumber ($cm^{-1}$) | Mode of vibration | Functional groups | References |
|---|---|---|---|
| 1700 | $\nu C{=}O$ | carbamic acid | 4-8 |
| 1658 | $\nu C{=}O$ | carbamic acid | 7 |
| 1650 - 1635 | $\delta_{as}NH_3^+$ | ammonium ion | 5, 8-11 |
| 1616 | $\nu_{as}COO^-$ | bicarbonate ion | 12 |
| 1580 | $\nu_{as}COO^-$ | carbamate ion | 7 |
| 1552 | $\nu_{as}COO^-$ | carbamate ion | 5-9 |
| 1537 | $\delta_{as}NH_3^+$ | ammonium ion | 7 |

| | | | |
|---|---|---|---|
| 1488 | $\nu COO^-$ | carbamate ion | 8-9, 13 |
| 1473 | $\delta_s NH_3^+$ | ammonium ion | 7 |
| 1435-1425 | $\nu_s COO^-$ | carbamate ion | 5-7,11 |
| 1410 | $\nu CN/NCOO^-$ skeletal vibration | carbamate ion | 8, 10 |
| 1380-1375 | $\nu_s COO^-$ | carbamate ion | 7 |
| 1358 | $\nu_s COO^-$ | bicarbonate ion | 6-7, 12 |
| 1320 | $NCOO^-$ skeletal vibration | carbamate ion | 6, 9 |

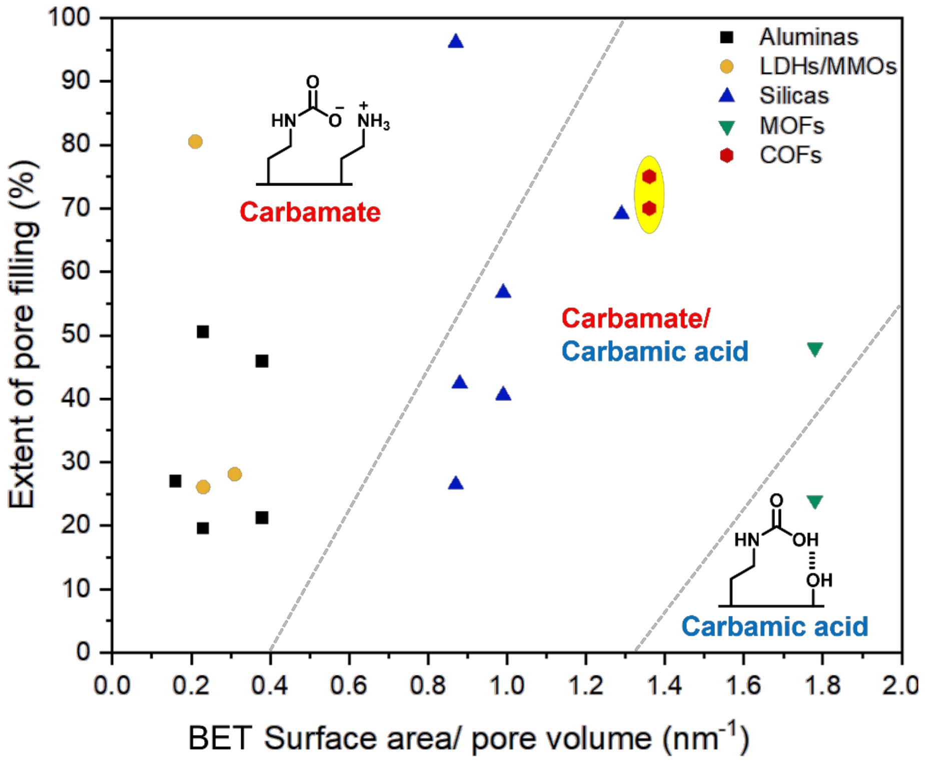


**Figure S23.** Extent of pore filling (%) vs. BET surface area/pore volume ($nm^{-1}$) plot presenting a 2D map of $CO_2$ capture mechanisms in solid-supported amine adsorbents, including alumina, silicas, LDHs/MMOs, MOFs, and COFs (this study: ImCOF-TAEA and ImCOF-TEPA, highlighted in yellow) under dry ambient DAC conditions. Depending on the pore characteristics and extent of pore filling by the amines, adsorption mechanism is dominated by carbamate formation, carbamic acid formation, or both. The figure is adapted from Ref. 14 with modifications, and data for alumina, silicas, LDHs/MMOs, and MOFs are taken from previous reports cited in Ref. 14.

### 4.6 Energy for Dry and Humid $CO_2$ Desorption from ImCOF-TAEA

The desorption energies of $CO_2$ from ImCOF-TAEA under dry and humid conditions at 25°C were estimated using the temperature programmed desorption (TPD) method reported by Cvetanović and Amenomiya,[15] based on the following equation:

$$2\ln(T_m) - \ln\beta = \frac{E_d}{RT_m} + ln\frac{E_d}{AR} \qquad \text{(Eq. 1)}$$

where $T_m$ is the peak maximum (corresponding to the peak position in the $CO_2$ TPD curve, Figure S22a, S22c), $\beta$ is the heating rate, $E_d$ is the desorption energy, A is the pre-exponential factor for desorption, and R is the universal gas constant (i.e., 8.314 J $mol^{-1}$ $K^{-1}$). Using experimentally determined $T_m$ values at different $\beta$, $E_d$ (kJ $mol^{-1}$) can be calculated. The regeneration energy of a sorbent in temperature swing adsorption is given by the sum of the heat of adsorption and the activation energy for desorption.

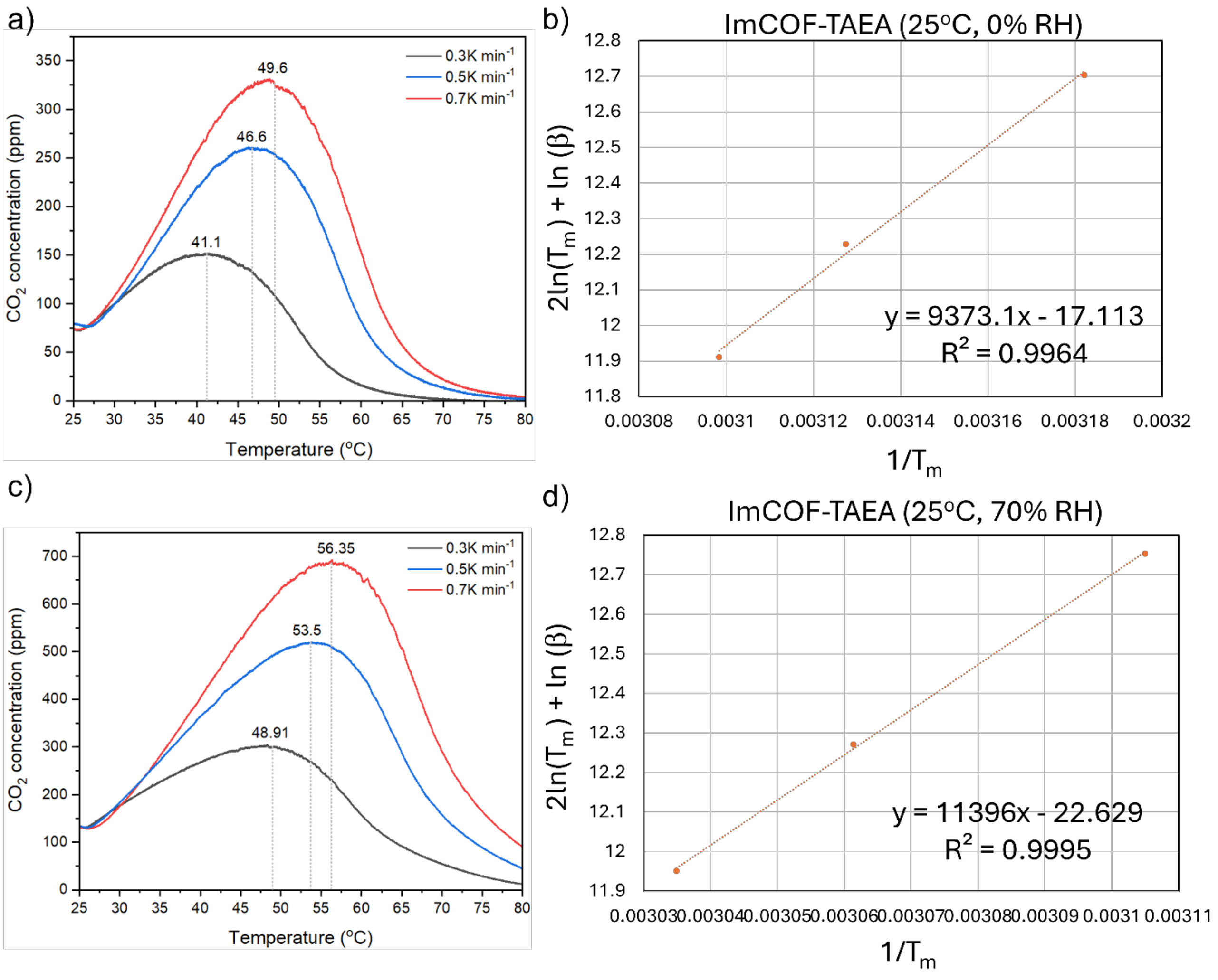


**Figure S24.** Temperature-programmed desorption (TPD) analysis of $CO_2$ adsorbed on ImCOF-TAEA at 25 °C under (a) dry and (c) 70% RH conditions, performed at heating rates of 0.3, 0.5, and 0.7 K $min^{-1}$. Energy of $CO_2$ desorption determined from equation (1) for (b) 0% RH (calculated $E_d$: 77.9 kJ $mol^{-1}$) and (d) 70% RH (calculated $E_d$: 94.8 kJ $mol^{-1}$) during 400 ppm $CO_2/N_2$ adsorption at 25ºC.

## 4.7 Summary of the Moisture-Enhanced $CO_2$ Capture Mechanism

Amine functionalized sorbents capture $CO_2$ through the formation of three primary species: alkylammonium carbamate ($R\text{-}NH_3^+\cdots{}^-OOC\text{-}NH\text{-}R$), carbamic acid (R-NH-COOH), and bicarbonate ($R\text{-}NH_3^+\cdots HCO_3^-$). The formation of these species follows distinct stoichiometric pathways:[16]

Ammonium carbonate: $R\text{-}NH_2 + CO_2 \rightleftharpoons R\text{-}NH_2^+COO^-$ ($E_a$ = ~40-50 kcal mol$^{-1}$)

$R\text{-}NH_2 + R\text{-}NH_2^+COO^- \rightleftharpoons R\text{-}NH\text{-}COO^- + R\text{-}NH_3^+$ ($E_a$ = ~40-50 kcal mol$^{-1}$)

Carbamic acid: $R\text{-}NH_2 + CO_2 \rightleftharpoons R\text{-}NH\text{-}COOH$ ($E_a$ = ~14-41 kcal mol$^{-1}$)

Bicarbonate: $R\text{-}NH_2 + CO_2 + H_2O \rightleftharpoons R\text{-}NH_3^+ + HCO_3^-$ ($E_a$ = ~21 kcal mol$^{-1}$)

Alkylammonium carbamate formation requires a 2:1 amine:$CO_2$ stoichiometry, leading to a theoretical maximum amine efficiency of 0.5 $mmol_{CO2}/mmol_N$. However, carbamic acid and bicarbonate formation follow a 1:1 amine:$CO_2$ stoichiometry, allowing for an efficiency of 1.0 $mmol_{CO2}/mmol_N$. The prevalence of each species depends on several factors, such as amine type, amine density, support pore structure, humidity, and $CO_2$ concentration.

In our previous studies, we demonstrated that the relative balance between amine loading and textural properties (surface area/pore volume ratio) strongly influences the mechanism of $CO_2$ binding (Figure S23).[4] High amine loading with low surface area/pore volume ratio favors ammonium carbamate formation. Whereas, low amine loading with high surface area/pore volume promotes carbamic acid formation. An intermediate domain supports coexistence of both pathways. The ImCOF-Amine materials fall within this intermediate region, consistent with our *in situ* DRIFTS results that show the formation of multiple chemisorbed species under dry $CO_2$ (Figure 5a, 5b, S23).

Upon transitioning from dry to humid conditions (70% RH), we observe a dramatic enhancement in $CO_2$ uptake a 2.4-fold increase at 25 °C, rising to 4.7-fold at -5 °C. This deviation from the expected stoichiometry under humid conditions (i.e., >2-fold increase) can be rationalized through the following considerations:

(i) Under dry conditions, strong intra- and inter-chain amine–amine H-bonding, and amine-support interactions restricts the mobility and accessibility of many amine sites (Figure 5e).[4,17-18] As a result, only a fraction of the total amines participates in $CO_2$ capture. In humid conditions, solvation by water molecules weakens these amine–amine interactions, increasing amine mobility and exposing previously inaccessible amines. This significantly increases the total number of reactive amines participating in $CO_2$ binding.

To qualitatively understand this effect, we estimated the corresponding accessible amine content from the TPD experiment (Figure S19). For the simplicity, we only consider the contributions from strong (associated with carbamate formation; 1:2 $CO_2$:amine binding) and weak (associated with carbamic acid/bicarbonate formation; 1:1 $CO_2$:amine binding) chemisorption. At 25 °C under dry conditions, the total $CO_2$ uptake is 0.46 mmol g$^{-1}$, comprising 0.31 mmol g$^{-1}$ strong and 0.15 mmol g$^{-1}$ weak chemisorption, corresponding to 0.77 mmol g$^{-1}$ accessible amines based on the respective stoichiometries (Figure 19c). Under 70% RH, these contributions increase to 0.86 mmol g$^{-1}$ (strong)

and 0.23 mmol $g^{-1}$ (weak), yielding 1.95 mmol $g^{-1}$ accessible amines, a 2.5-fold increase relative to dry conditions. At 25 °C and 30% or 50% RH, the accessible amine contents rise to 0.90 mmol $g^{-1}$ and 1.32 mmol $g^{-1}$, respectively. At -5 °C and 70% RH, the amines reacting with $CO_2$ total 1.67 mmol $g^{-1}$, slightly reduced due to lower chain mobility at sub-ambient temperatures.

(ii) Formation of ammonium carbamate requires cooperative interactions between pairs of amines. Under dry conditions and especially at low temperatures, restricted chain mobility limits the ability of amines to adopt these favorable geometries. The presence of water enhances chain flexibility, enabling amines to more readily achieve the required conformations, thus increasing the overall capacity beyond what dry-state stoichiometry would predict. In addition, water acts as a proton acceptor (Brønsted base) and facilitates proton transfer processes, thereby promoting the formation of ammonium carbamate ion pairs. Notably, Jung and coworkers demonstrated through DFT calculations that the activation energy for alkylammonium carbonate formation in the presence of water (6.0 kcal $mol^{-1}$) is less than half of that required under dry conditions (12.7 kcal $mol^{-1}$).[19-20]

(iii) Humidity favors reactions that involve proton transfer and stabilization by water, especially the formation of carbamic acid, and bicarbonate. Formation of these species follows a 1:1 stoichiometry, thereby doubling the theoretical $CO_2$ loading relative to ammonium carbamate. This shift in mechanism further contributes to the observed efficiency under humid conditions.

Therefore, we hypothesized that the large enhancement in $CO_2$ uptake under humid conditions cannot be attributed solely to the switch from carbamate to carbamic acid/bicarbonate chemistry as observed from DRIFTS analysis. Rather, the dominant factor is the water-induced increase in amine accessibility, enabling a greater fraction of the amines to participate in $CO_2$ binding, regardless of the specific reaction pathway.

**4.8 Recyclability of ImCOF-TAEA**

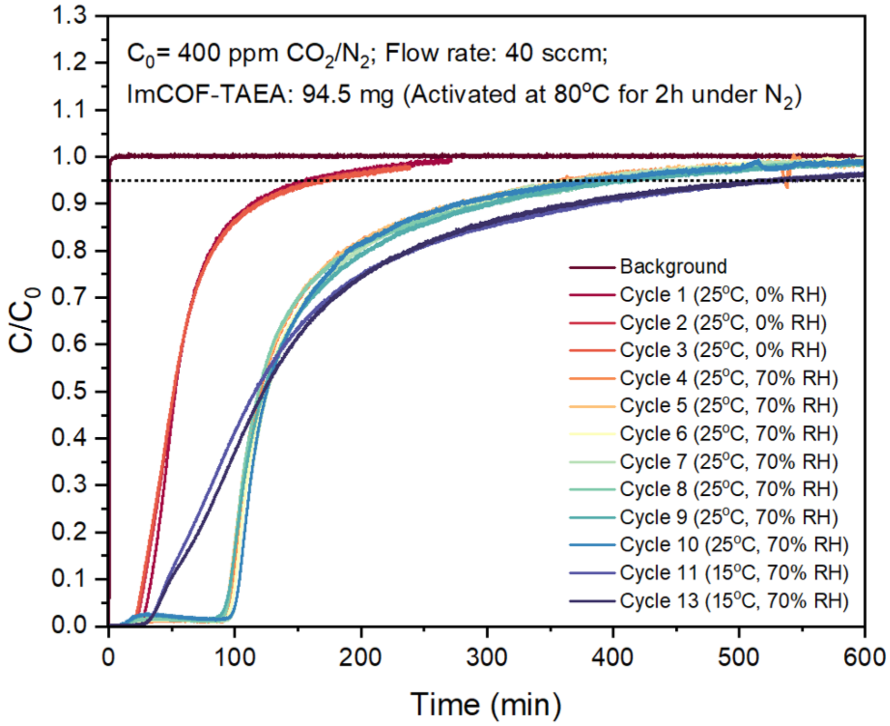


**Figure S25**. Breakthrough curves showing the recyclability of ImCOF-TAEA under DAC conditions at 25°C with 0% and 70% RH, and at 15°C with 70% RH. Adsorption was carried out using dry/humid ~400 ppm $CO_2/N_2$ (co-adsorption) at a flow rate of 40 sccm, and each run was terminated when the outlet $CO_2$ concentration reached 95% of the inlet concentration ($C_0$). Regeneration of ImCOF-TAEA was performed after each adsorption cycle at 80°C under $N_2$ flow (60 sccm) for 2 h.

The observed fluctuation in breakthrough capacity is primarily attributed to kinetic and mass-transfer limitations rather than irreversible loss of active sites (Figure 6b, S25). This behavior likely originates from conformational rearrangements of amine chains during repeated adsorption-desorption cycles, driven by amine-amine interactions and transient cross-linking associated with ammonium carbonate formation.[21-22] Importantly, the stable pseudo-equilibrium capacity demonstrates that the framework and amine functionalities remain intact, confirming that the reduced uptake under 0% RH conditions is reversible and governed by kinetic effects rather than structural or chemical degradation.

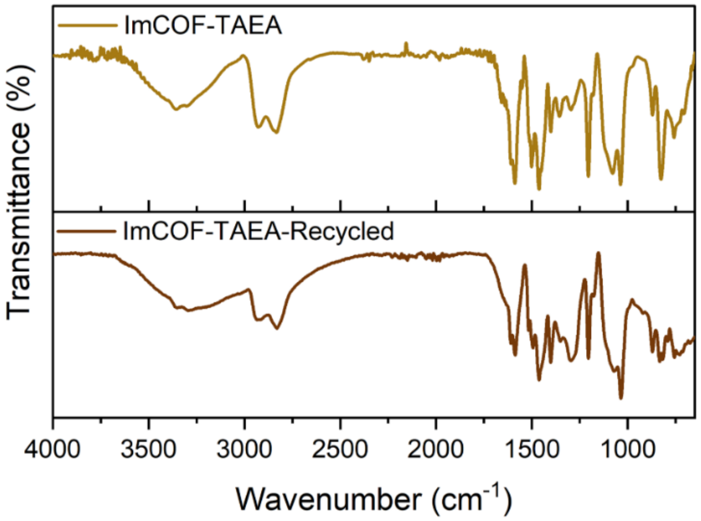


**Figure S26.** FTIR spectra of ImCOF-TAEA before (top) and after recycling (bottom) show that the characteristic stretching bands at ~2930 and ~2830 cm$^{-1}$ (aliphatic C-H) and ~3350 cm$^{-1}$ (N-H from grafted TAEA) remain nearly unchanged, confirming the preservation of the amine functionalities after recycling. The overall spectral similarity indicates that the structural framework of the COF remains stable during the recycling process. A change observed near 1260 cm$^{-1}$ may arise from subtle changes in C-O linkages within the COF backbone, rather than any significant structural degradation.

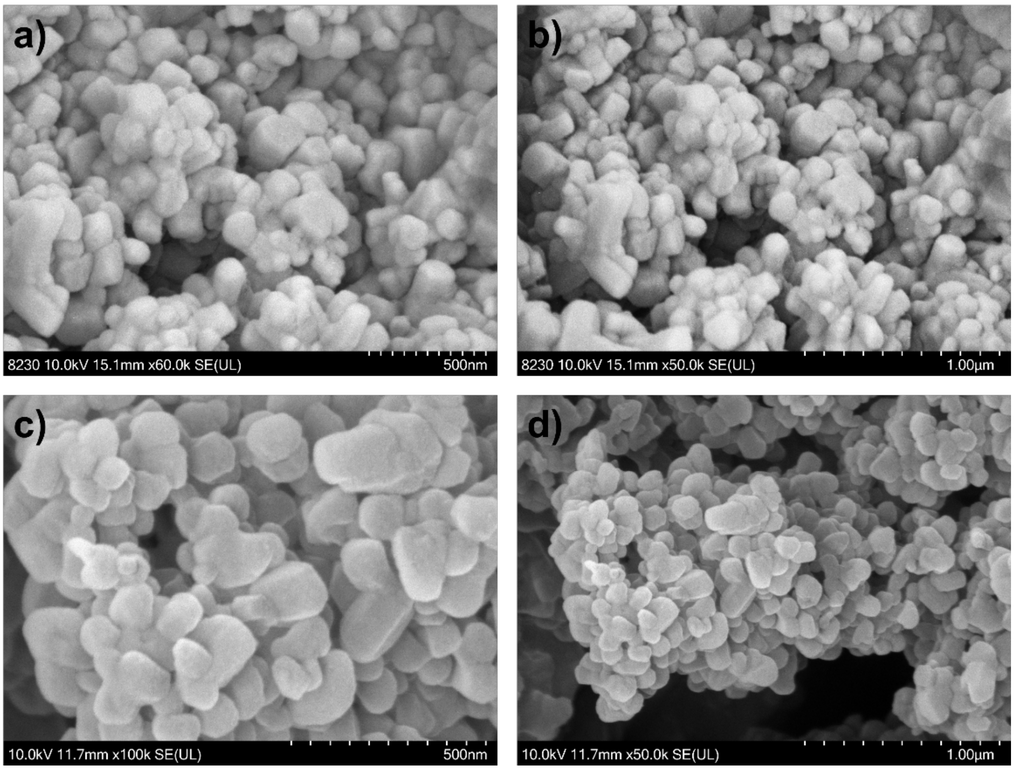


**Figure S27.** FESEM images of ImCOF-TAEA (a), (b) before (top panel) and (c), (d) after recycling (bottom panel).

## 5. Comparative Table

**Table S4.** Comparative table based on the direct air capture performance of benchmark amine-grafted solid sorbents under dry and humid conditions.

| Entry | Amine-grafted Adsorbent | $CO_2$ Capacity under DAC Conditions | | Enhancement in $CO_2$ Capacity (%) from Dry to Humid | Reference |
|---|---|---|---|---|---|
| | | Dry | Humid | | |
| 1 | ImCOF-TAEA | 0.46 ± 0.02 | 1.09 ± 0.09 (70% RH) | 137% at 25°C | *Present work* |
| | | 0.41 | 1.25 ± 0.02 (70% RH)<br>1.52 (70% RH, presaturation) | 205% at 15°C<br>271% (presaturation, at 15°C) | |
| | ImCOF-TEPA | 0.21 | 0.86 (70% RH) | 310% at 25°C | |
| **Covalent Organic Frameworks (COFs)** | | | | | |
| 2 | COF-609 | 0.30 | 0.39 (50% RH) | 29% at 25°C | 2 |
| 3 | COF-709 | 0.48 | 1.24 (75% RH) | 158% at 25°C | 23 |
| 4 | COF-999 | 0.96 | 2.09 (75% RH) | 118 % at 25°C | 3 |
| **Metal Organic Frameworks (MOFs)** | | | | | |
| 5 | MOF-808-Lys | 0.612 | 1.205 (50% RH) | 97% at 25°C | 24 |
| 6 | MOF-808-TAPA | 0.498 | 0.872 (50% RH) | 75% at 25°C | |
| 7 | MIL-101(Cr)-TEPA (30wt%) | 0.11 | 0.38 (70% RH) | 245% at 25°C | 4 |
| 8 | NUS-110 | 0.89 | 1.31 (30% RH),<br>0.34 (80% RH) | 47% at 25°C for 30% RH; -62% for 80% RH | 25 |
| **Resins** | | | | | |
| 9 | PPVAm-3.3 | 0.9 | 0.95 (50% RH) | 5.5% at 25°C | 26 |
| 10 | PEI-HP20 | 1.25 | 1.35 (50% RH) | 8% at 25°C | 27 |
| 11 | Purolite A110 | 1.30 | 1.76 (33% RH) | 35.4% at 25°C | 28 |
| **Silica** | | | | | |
| 12 | DT- PE-MCM-41 | 0.61 | 0.54 (73% RH) | -11.5% at 25°C | 29 |
| 13 | AEAPTMS-SBA-15 | 0.98 | 1.72 (100% RH) | 75.5% at 25°C | 30 |
| 14 | ED-Silica gel | 0.40 | 0.44 (40% RH) | 10% at 25°C | 31 |
| **Alumina** | | | | | |
| 15 | TRI-PE-MCM-41 | 0.98 | 1.53 (64% RH) | 56% at 25°C | 32 |
| 16 | TRI-Alumina | 0.48 | 0.74 (50% RH) | 54% at 25°C | 33 |

**N.B.:** The present table exclusively includes "chemically grafted polyamines" on porous supports, as these systems are relevant to the current study. The table specifically highlights humidity-enhanced direct $CO_2$ capture performance. Other classes of $CO_2$ sorbents, such as physically impregnated amines, liquid amines, ionic liquids, or small molecular amines, are not included, since their material characteristics and some of the cases adsorption mechanisms differ fundamentally from the covalently functionalized systems considered here.

## 6. References


(1) Xu, H.; Tao, S. S.; Jiang, D. L. Proton Conduction in Crystalline and Porous Covalent Organic Frameworks. *Nat. Mater.* **2016**, *15*, 722–726.

(2) Lyu, H.; Li, H. Z.; Hanikel, N.; Wang, K. Y.; Yaghi, O. M. Covalent Organic Frameworks for Carbon Dioxide Capture from Air. *J. Am. Chem. Soc.* **2022**, *144*, 12989–12995.

(3) Zhou, Z. H.; Ma, T. Q.; Zhang, H. Y.; Chheda, S.; Li, H. Z.; Wang, K. Y.; Ehrling, S.; Giovine, R.; Li, C. S.; Alawadhi, A. H.; Abduljawad, M. M.; Alawad, M. O.; Gagliardi, L.; Sauer, J.; Yaghi, O. M. Carbon Dioxide Capture from Open Air using Covalent Organic Frameworks. *Nature* **2024**, *635*, 96–101.

(4) Rim, G.; Priyadarshini, P.; Song, M.; Wang, Y.; Bai, A.; Realff, M. J.; Lively, R. P.; Jones, C. W. Support Pore Structure and Composition Strongly Influence the Direct Air Capture of $CO_2$ on Supported Amines. *J. Am. Chem. Soc.* **2023**, *145*, 7190–7204.

(5) Bacsik, Z.; Ahlsten, N.; Ziadi, A.; Zhao, G. Y.; Garcia-Bennett, A. E.; Martin-Matute, B.; Hedin, N. Mechanisms and Kinetics for Sorption of $CO_2$ on Bicontinuous Mesoporous Silica Modified with n-Propylamine. *Langmuir* **2011**, *27*, 11118–11128.

(6) Didas, S. A.; Salcwa-Novak, M. A.; Foo, G. S.; Sievers, C.; Jones, C. W. Effect of Amine Surface Coverage on the Co-Adsorption of $CO_2$ and Water: Spectral Deconvolution of Adsorbed Species. *J. Phys. Chem. Lett.* **2014**, *5*, 4194–4200.

(7) Foo, G. S.; Lee, J. J.; Chen, C. H.; Hayes, S. E.; Sievers, C.; Jones, C. W. Elucidation of Surface Species through in Situ FTIR Spectroscopy of Carbon Dioxide Adsorption on Amine-Grafted SBA-15. *ChemSusChem* **2017**, *10*, 266–276.

(8) Wilfong, W. C.; Srikanth, C. S.; Chuang, S. S. C. *ACS Appl. Mater. Interfaces* **2014**, *6*, 13617–13626.

(9) Yu, J.; Chuang, S. S. C. The Structure of Adsorbed Species on Immobilized Amines in $CO_2$ Capture: An *In Situ* IR Study. *Energy Fuels* **2016**, *30*, 7579–7587.

(10) Wang, X. X.; Schwartz, V.; Clark, J. C.; Ma, X. L.; Overbury, S. H.; Xu, X. C.; Song, C. Infrared Study of $CO_2$ Sorption over "Molecular Basket" Sorbent Consisting of Polyethylenimine-Modified Mesoporous Molecular Sieve. *J. Phys. Chem. C* **2009**, *113*, 7260–7268.

(11) Danon, A.; Stair, P. C.; Weitz, E. FTIR Study of $CO_2$ Adsorption on Amine-Grafted SBA-15: Elucidation of Adsorbed Species. *J. Phys. Chem. C* **2011**, *115*, 11540–11549.

(12) Lee, J. J.; Chen, C.-H.; Shimon, D.; Hayes, S. E.; Sievers, C.; Jones, C. W. Effect of Humidity on the $CO_2$ Adsorption of Tertiary Amine Grafted SBA-15. *J. Phys. Chem. C* **2017**, *121*, 23480–23487.

(13) Tumuluri, U.; Isenberg, M.; Tan, C. S.; Chuang, S. S. C. In Situ Infrared Study of the Effect of Amine Density on the Nature of Adsorbed $CO_2$ on Amine-Functionalized Solid Sorbents. *Langmuir* **2014**, *30*, 7405–7413.

(14) Rim, G.; Song, M.; Proano, L.; Nik, O. G.; Parker, S.; Lively, R. P.; Jones, C. W. Humidity Effects on Sub-Ambient Direct Air Capture of $CO_2$ with Amine Functionalized Mg-Al LDHs and MMOs. *ACS EST Eng.* **2025**, *5*, 204–214.

(15) Cvetanović, R. J.; Amenomiya, Y. In *Advances in Catalysis*; Eley, D. D.; Pines, H.; Weisz, P. B., Eds.; Academic Press: New York, 1967; Vol. 17, pp 103–149.

(16) Ryu, U.; Min, Y. J.; Zhao, W.; Lee, Y.; Realff, M. J.; Jones, C. W. Climatic Conditions and Amine Loading Impact the Performance of Laminate-Supported Poly(ethylenimine) Direct Air Capture Sorbents. *JACS Au* 2025, doi.org/10.1021/jacsau.5c01294.

(17) Song, M. G.; Rim, G.; Mirzazadeh, G.; Hoffman, J.; Moon, H. J.; Leisen, J. E.; Nik, O. G.; Lively, R. P.; Jones, C. W. Amine-Dependent $CO_2$ Sorption on Amine-impregnated Mg(dobpdc) MOF under Humid Conditions. *Ind. Chem. Mater.* **2025**, doi.org/10.1039/D5IM00002E.

(18) Sardo, M.; Bordonhos, M.; Afonso, R.; Juzkow, J.; Pinto, M. L.; Pacheco, M.; Gomes, J.; Mafra, L. Unravelling Moisture-induced $CO_2$ Chemisorption Mechanisms in Amine-modified Sorbents at the Molecular Scale. *J. Mater. Chem. A* **2021**, *9*, 5542-5555.

(19) Cho, M.; Park, J.; Yavuz, C. T.; Jung, Y. A Catalytic Role of Surface Silanol Groups in $CO_2$ Capture on the Amine-Anchored Silica Support. *Phys. Chem. Chem. Phys.* **2018**, *20*, 12149-12156.

(20) Kolle, J. M.; Fayaz, M.; Sayari, A. Understanding the Effect of Water on $CO_2$ Adsorption. *Chem. Rev.* **2021**, *121*, 7280−7345.

(21) Holewinski, A.; Sakwa-Novak, M. A.; W. Jones, C. W. Linking $CO_2$ Sorption Performance to Polymer Morphology in Aminopolymer/Silica Composites through Neutron Scattering. *J. Am. Chem. Soc.* **2015**, *137*, 11749–11759.

(22) Lee, J. J.; Yoo, C. J.; Chen, C. H.; Hayes, S. E.; Sievers, C.; Jones, C. W. Silica-Supported Sterically Hindered Amines for $CO_2$ Capture. *Langmuir* **2018**, *34*, 12279–12292.

(23) Li, H. Z.; Zhou, Z. H.; Ma, T. Q.; Wang, K. Y.; Zhang, H. Y.; Alawadhi, A. H.; Yaghi, O. M. Bonding of Polyethylenimine in Covalent Organic Frameworks for $CO_2$ Capture from Air. *J. Am. Chem. Soc.* **2024**, *146*, 35486–35492.

(24) Chen, O. I.-F.; Liu, C.-H.; Wang, K.; Borrego-Marin, E.; Li, H.; Alawadhi, A. H.; Navarro, J. A. R.; Yaghi, O. M. Water-Enhanced Direct Air Capture of Carbon Dioxide in Metal–Organic Frameworks. *J. Am. Chem. Soc.* **2024**, *146*, 2835–2844.

(25) Ren, J.; Zhang, Z.; Zhu, N.; Yu, K.; Shi, X.; Li, H.; Liu, Q.; Joshi, Y. V.; Abubakar, S.; Zhao, D. Bioinspired Reconfiguration of Alkylamines into Metal−Organic Frameworks for Robust Direct Air Capture. *J. Am. Chem. Soc.* **2025**, *147*, 43517−43528.

(26) Baker, J.; Muldoon, P.; Steckel, J. A.; Sekizkardes, A. K. Porous Polyvinylamine Adsorbents with Low Temperature Regenerability for Direct Air Capture. *ACS Appl. Polym. Mater.* **2024**, *6*, 14278–14282.

(27) Wang, Y.; Li, G. K. The Impact of Co-adsorbed Water on Energy Consumption and $CO_2$ Productivity in Direct Air Capture Systems. *Sep. Purif. Technol.* **2025**, *354*, 129415.

(28) Low, M.-Y. A.; Danaci, D.; Azzan, H.; Woodward, R. T.; Petit, C. Measurement of Physicochemical Properties and $CO_2$, N2, Ar, O2, and H2O Unary Adsorption Isotherms of Purolite A110 and Lewatit VP OC 1065 for Application in Direct Air Capture. *J. Chem. Eng. Data* **2023**, *68*, 3499–3511.

(29) Wagner, A.; Steen, B.; Johansson, G.; Zanghellini, E.; Jacobsson, P.; Johansson, P. Carbon Dioxide Capture from Ambient Air Using Amine-Grafted Mesoporous Adsorbents. *Int. J. Spectrosc.* **2013**, *2013*, 1–8. https://doi.org/10.1155/2013/690186.

(30) Choi, S.; Drese, J. H.; Eisenberger, P. M.; Jones, C. W. Application of Amine-Tethered Solid Sorbents for Direct $CO_2$ Capture from the Ambient Air. *Environ. Sci. Technol.* **2011**, *45*, 2420–2427.

(31) Wurzbacher, J. A.; Gebald, C.; Steinfeld, A. Separation of $CO_2$ from Air by Temperature-Vacuum Swing Adsorption using Diamine-functionalized Silica Gel. *Energy Environ. Sci.* **2011**, *4*, 3584–3592.

(32) Belmabkhout, Y.; Serna-Guerrero, R.; Sayari, A. Adsorption of $CO_2$-Containing Gas Mixtures over Amine-Bearing Pore-Expanded MCM-41 Silica: Application for Gas Purification. *Ind. Eng. Chem. Res.* **2010**, *49*, 359–365.

(33) Grossmann, Q.; Saenz-Cavazos, P. A.; Ferru, N.; Williams, D. R.; Mazzotti, M. Measuring and Modeling Water and Carbon Dioxide Adsorption on Amine Functionalized Alumina under Direct Air Capture Conditions. *Ind. Eng. Chem. Res.* **2025**, *64*, 7165–7175.
